# The Discovery of Imine Reductases and their Utilisation for the Synthesis of Tetrahydroisoquinolines

**DOI:** 10.1002/cctc.202201126

**Published:** 2023-01-11

**Authors:** Max Cárdenas‐Fernández, Rebecca Roddan, Eve M. Carter, Helen C. Hailes, John M. Ward

**Affiliations:** ^1^ Department of Biochemical Engineering University College London Gower Street, Bernard Katz Building London WC1E 6BT UK; ^2^ School of Biosciences University of Kent K ent CT2 7NJ UK; ^3^ Department of Chemistry University College London 20 Gordon Street London WC1H 0AJ UK

**Keywords:** Imine reductases, reductive amination, activity screening, phylogenetic analysis, tetrahydroisoquinolines

## Abstract

Imine reductases (IREDs) are NADPH‐dependent enzymes with significant biocatalytic potential for the synthesis of primary, secondary, and tertiary chiral amines. Their applications include the reduction of cyclic imines and the reductive amination of prochiral ketones. In this study, twenty‐nine novel IREDs were revealed through genome mining. Imine reductase activities were screened at pH 7 and 9 and in presence of either NADPH or NADH; some IREDs showed good activities at both pHs and were able to accept both cofactors. IREDs with Asn and Glu at the key 187 residue showed preference for NADH. IREDs were also screened against a series of dihydroisoquinolines to synthesise tetrahydroisoquinolines (THIQs), bioactive alkaloids with a wide range of therapeutic properties. Selected IREDs showed high stereoselectivity, as well high THIQ yields (>90 %) when coupled to a glucose‐6‐phosphate dehydrogenase for NADPH cofactor recycling.

## Graphical abstract

**Twenty‐nine novel Imine reductases (IREDs)** were mined from 9 microorganisms, and their activity screened against model substrates and at different conditions. IREDs were successfully used for the synthesis of tetrahydroisoquinolines (THIQs), bioactive alkaloids with a wide range of therapeutic properties.

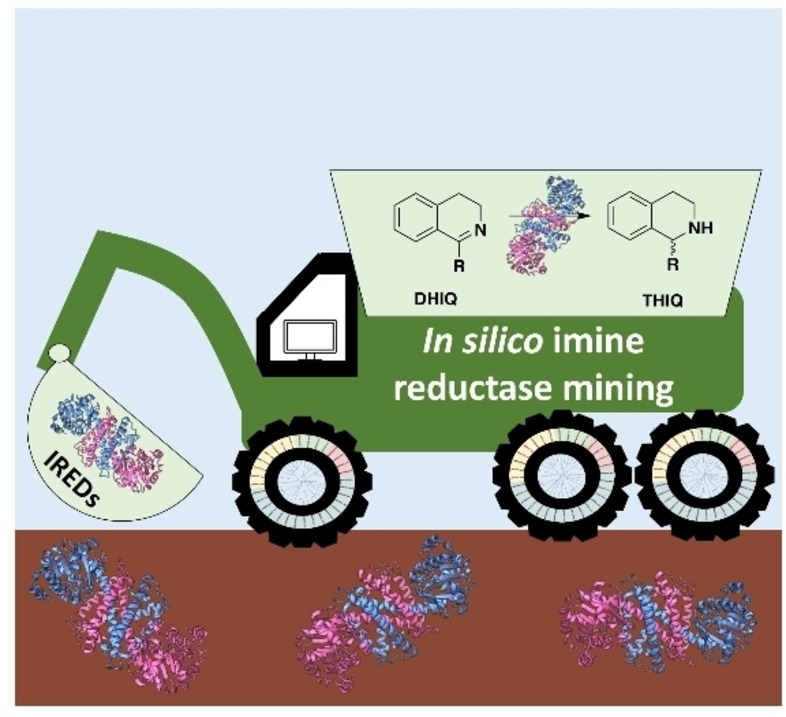

## Introduction

**Scheme 1:**
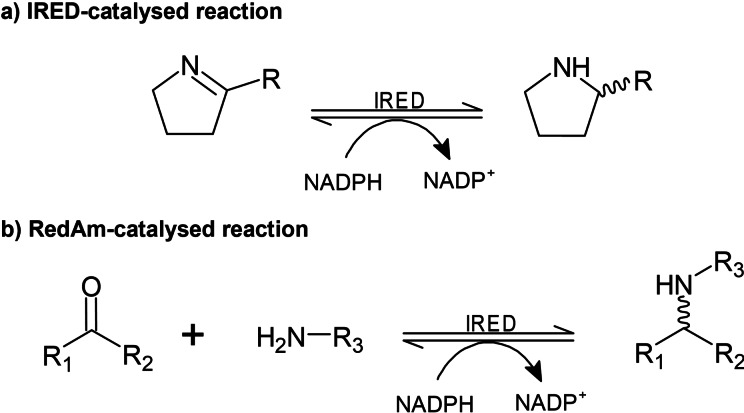
The biocatalytic reduction of imines catalysed by IREDs.

Chiral amines play an important role in the synthesis of bioactive compounds for the fine chemical, pharmaceutical and agrochemical industries and it is estimated that around 40 % of pharmaceutical drugs contain a chiral amine component in their structure.^[1]^ Due to the high stereoselectivities that can be achieved, several sustainable biocatalytic routes are currently used in industrial processes for chiral amine synthesis involving biocatalysts such as lipases,^[2]^ transaminases,^[3]^ ammonia lyases,^[4]^ amine dehydrogenases^[5]^ and amine oxidases.^[6]^ Imine reductases (IREDs) are a relatively new group of enzymes that were first reported in 2010 for reduction of the imine 2‐methyl‐1‐pyrroline into (*R*) or (*S*)‐2‐methylpyrrolidine.^[7]^ IREDs belong to the NADPH‐dependent oxidoreductase enzyme class and have a preference for the reduction of cyclic imines into chiral amines (Scheme 1a).[^8^, ^9^] They are less efficient at achieving the intermolecular reductive amination of prochiral ketones with amines but recently a subclass of IREDs, reductive aminases (RedAms), have been reported to convert these via imines into the respective amines (Scheme 1b).^[10]^ In addition, it has also been reported that IREDs can catalyse the kinetic resolution of chiral secondary amines in the presence of NADP^+^ at high pH values.^[11]^ For these reasons, IREDs are considered promising and powerful biocatalysts for the synthesis of a broad range of chiral primary, secondary and tertiary amine products; and a valuable alternative to other biocatalysts currently used for this purpose.

Traditional synthetic routes to access the same products afforded by IRED‐catalysed reactions can be challenging to achieve in high stereoselectivities, and reduction of the carbonyl group can lead to side products. The use of biocatalysis therefore offers an exciting opportunity for industrial‐scale chiral amine syntheses. However, some drawbacks for the wider application of IREDs remain such as poor activity, low stability, and narrow substrate specificity (especially (*R*)‐selective IREDs).[^12^, ^13^] Recently, significant effort has been made to discover new IREDs from plants, bacteria and fungi,[^14^, ^15^, ^16^, ^17^] as well as from metagenomes,[^18^, ^19^, ^20^] together with studies to better understand this enzyme class from a structural, mechanistic and biochemical perspective.

Recent reviews have highlighted the application of IREDs in the synthesis of several drugs and pharmaceutical intermediates, including alkaloids such as tetrahydroisoquinolines (THIQs).[^21^, ^22^] Many THIQs are naturally synthesised by plants and the scaffold has an important role in medicinal chemistry due to the wide range of pharmacological properties exhibited, such as anti‐HIV, antitumor, anti‐inflammatory, and use as bronchodilators and antibiotics.[^23^, ^24^, ^25^, ^26^] Different biocatalytic routes to access THIQs have been studied using monoamine oxidases, norcoclaurine synthases and the berberine bridge enzyme.[^22^, ^23^, ^24^, ^25^, ^26^, ^27^, ^28^] IREDs have been described for the asymmetric reduction of dihydroisoquinolines (DHIQs) to chiral THIQs, being more advantageous compared to chemical reduction methods where enantioselectivity and cost still remain challenging.[^29^, ^30^, ^31^]

This work aims to expand the number of IREDs through a genome mining approach from nine bacterial strains; their IRED activities were screened against 2‐methyl‐1‐pyrroline, cyclohexanone and 2‐hexanone together with methylamine, at pH 7 and 9, and in presence of NADPH and NADH. In addition, IRED activity on DHIQs towards THIQs synthesis was examined, and to increase THIQ reaction yields, selected IREDs were coupled with a glucose‐6‐phosphate dehydrogenase (G6PDH) for cofactor recycling.

## Results and Discussion

### Uncovering novel IREDs

IREDs were mined from nine different microorganisms mainly from the *Streptomyces* genus. IREDs are mainly found in these strains, which is associated with their ability to produce various secondary metabolites such as antibiotics, peptides, pigments and bioherbicides, that contain amine functional groups.^[32]^ Protein sequences of putative (*S*)‐ and (*R*)‐IREDs were retrieved from BLASTp using the sequence of an IRED from *S. kanamyceticus* as a model protein (Uniprot Q1EQE0)^[33]^ against the whole genome of the specific microorganism.

Sequences were then confirmed by identifying two IRED‐specific motifs: first, the NADPH‐cofactor binding motif GLGxMGx5[ATS]x4Gx4[VIL]WNR[TS]x2[KR]; and second, the active site motif Gx[DE]x[GDA]x[APS]x3{K}x[ASL]x[LMVIAG]. Sequences with N191 were excluded as being one of the major determinants that differentiate IREDs from β‐hydroxyacid dehydrogenases.^[34]^ Twenty‐nine IREDs were uncovered following this *in silico* strategy, then cloned via our one‐pot restriction‐ligation reaction method^[35]^ using *E. coli* BL21(DE3) or *E. coli* Rosetta 2(DE3) as the expression host (see Materials and Methods). pQR numbers (the Ward group plasmid identifier) were consecutively assigned from pQR2595 to pQR2623 (see protein IDs in Supporting Information).

### IREDs sequence and phylogenetic analyses

**Figure 1:**
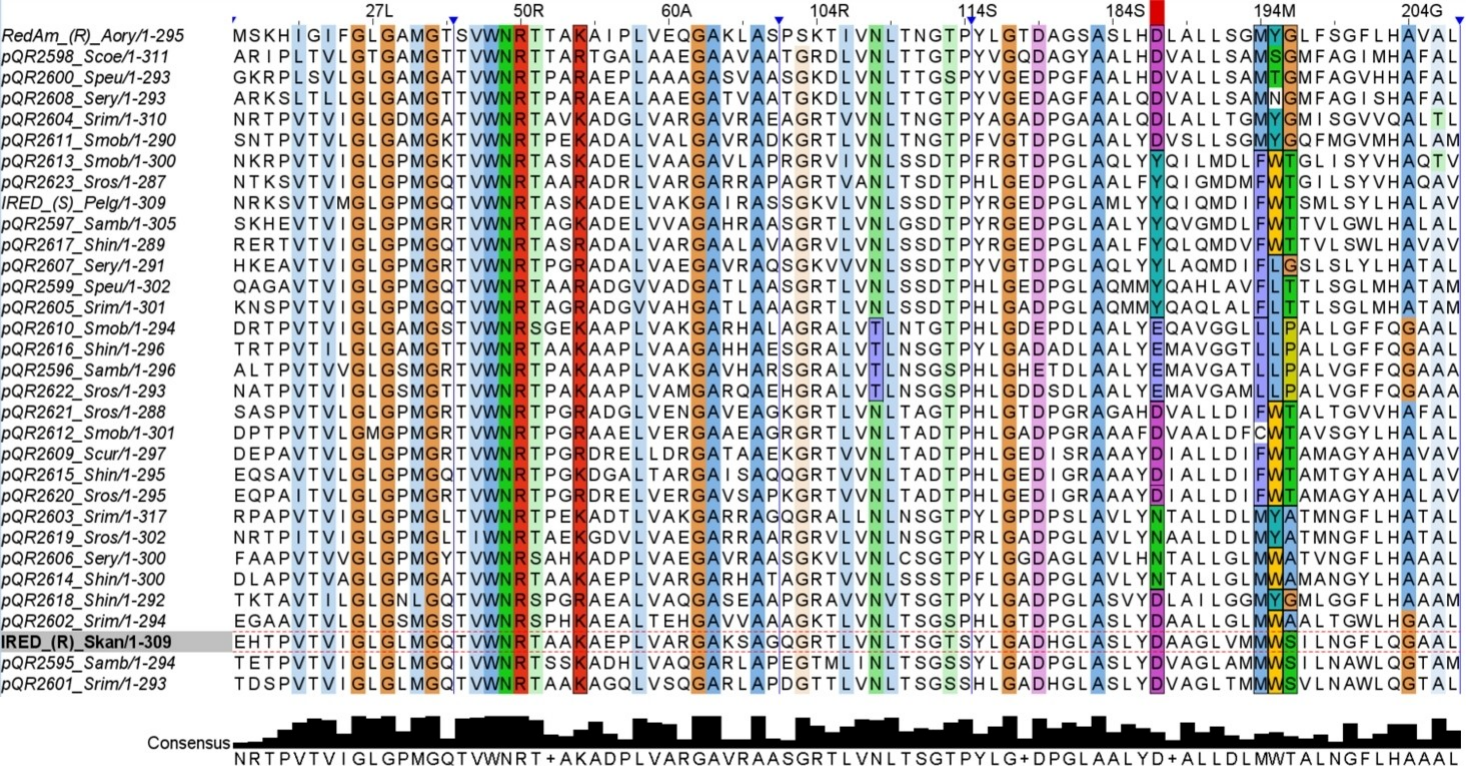
Partial sequence alignment was generated using Clustal Omega and analysed with Jalview bioinformatic software, including twenty‐nine novel IREDs, *S. kanamyceticus* ((R)‐IRED_Skan, Q1EQE0, set as numbering reference), (R)‐IRED *Aspergillus oryzae* and (S)‐IRED *Paenibacillus elgii* (Pelg, WP_010497949.1).

**Figure 2:**
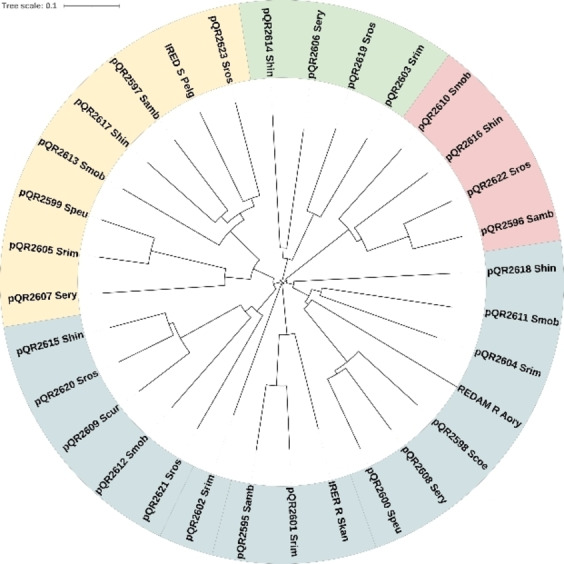
Phylogenetic analysis was generated with Clustal Omega and formatted using the iTOL server (itol.embl.de/index.shtml), including twenty‐nine novel IREDs, *S. kanamyceticus* ((*R*)‐IRED_Skan, Q1EQE0, (*R*)‐IRED *Aspergillus oryzae* (Aory, Q2TW47) and (*S*)‐IRED *Paenibacillus elgii* (Pelg, WP_010497949.1). D187 subgroup, blue; Y187 subgroup, yellow; N187 subgroup, green; and E187 subgroup, red.

Multiple sequence alignment of the IREDs was performed using the Clustal Omega online tool, including protein sequences of well‐characterised IREDs with known stereoselectivities: (*R*)‐IRED_Skan (set as numbering reference), (*R*)‐IRED_Aory *(Aspergillus oryzae*, Q2TW47) and (*S*)‐IRED_Pelg (*Paenibacillus elgii*, WP_010497949.1) (Figure 1). The sequence similarity of the IREDs ranged from 29 % to 71 % . Regarding the cofactor binding site, all IREDs contained a full‐length Rossmann‐fold domain GxGxxG consensus sequence and a highly conserved VWNR sequence (except pQR2619 with IWNR) (Figure 1). Key amino acid residues in the active site, D187 or Y187, are associated with the (*R*)‐ and (*S*)‐stereoselectivity, respectively; although this in not fully understood until now.^[17]^ Twenty‐one IREDs had one of these residues in this position, whilst some IREDs showed either E187 (pQR2596, 2610, 2616 and 2622) or N187 (pQR2603, 2606, 2614 and 2619) (Figure 1). The presence of E187 has been reported previously, with this residue acting as a proton donor during catalysis (similar to D187); however, N187 is not commonly found in IREDs. In fact, IREDs can be grouped based on this amino acid residue, as depicted in the phylogenetic tree (Figure 2), with E187 being more closely related to the D187 subgroup, and N187 related to the Y187 subgroup.

In addition, an interesting amino acid conservation pattern was observed in position 196 that is coincidentally found in each subgroup in the phylogenetic tree, which allows us to sub‐divide these IREDs into 8 additional subgroups, as summarised in Table 1. The D187 subgroup (pQR2598, 2600, 2604, 2608 and 2611) including (*R*)‐IRED_Aory (first RedAm to be reported)^[15]^ has a highly conserved G196; all Y187 (except pQR2607) and some D187 IREDs (subgroup pQR2609, 2612, 2615, 2620 and 2621), are closely related and show a conserved T196. Likewise, E187 and N187 show highly conserved P196 and A196, respectively; the remaining D187 IREDs (pQR2595, 2601 and IRED_Skan) show conserved S196, except pQR2618 with G199 and pQR2602 with A196.

**Table 1:** IREDs subgroups based on key amino acid residues 187 and 196 ((*R*)‐IRED_Skan, Q1EQE0, set as numbering reference).

Amino acid residue		pQR number
187	196	
D	G	2598, 2600, 2604, 2608, 2611, 2618
Y	T	2597, 2599, 2605, 2613, 2617, 2623
Y	G	2607
E	P	2596, 2610, 2616, 2622
D	T	2609, 2612, 2615, 2620, 2621
N	A	2603, 2606, 2614, 2619
D	A	2602
D	S	2595, 2601

Moreover, N108 was highly conserved amongst all these IREDs except for E187 IREDs containing T108 instead. These specific amino acid residues might play a significant role either in stereoselectivity or activity; however, this is beyond the aim of our study and would need to be proved experimentally.

### IREDs activity screening

**Scheme 2:**
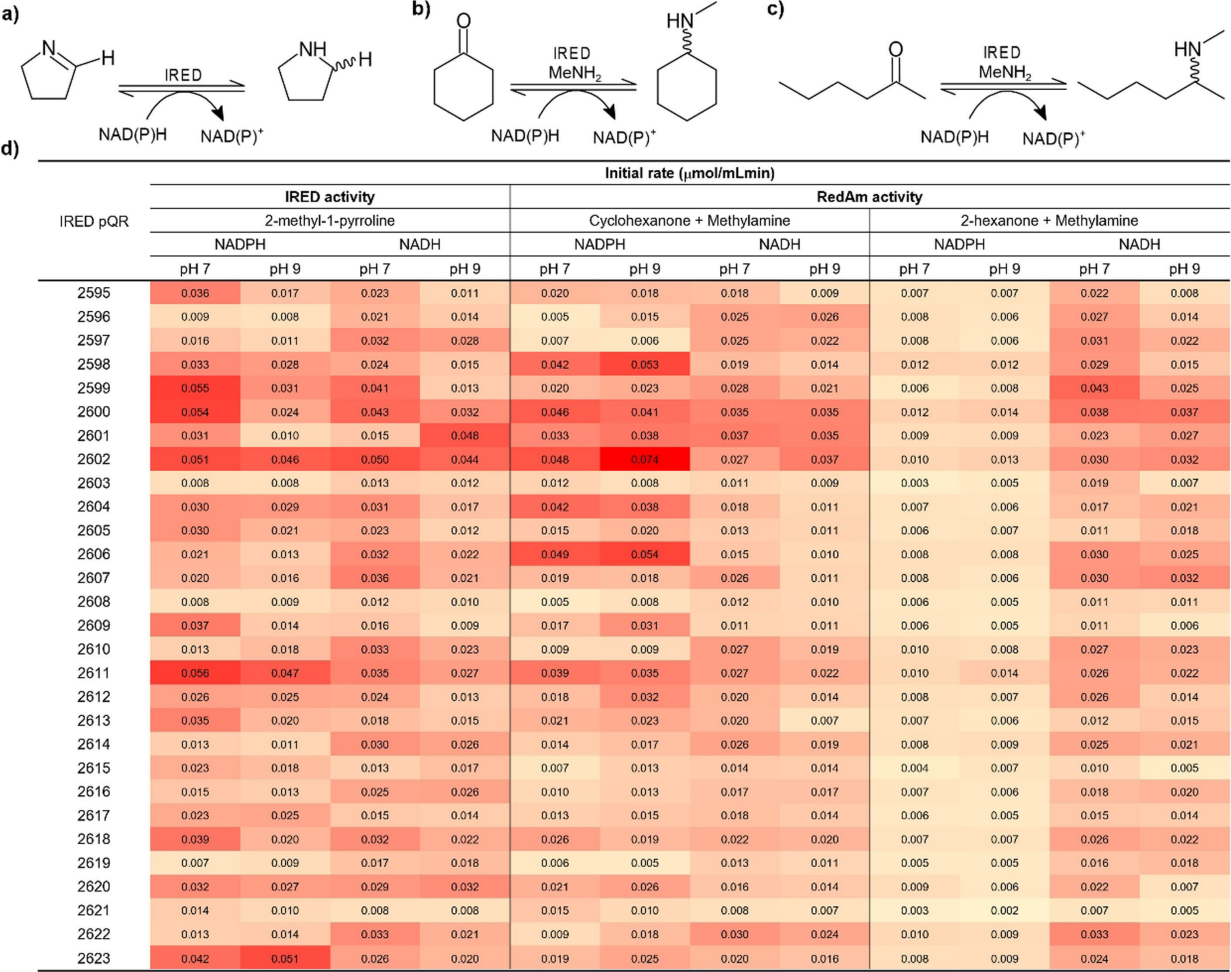
IREDs screening activity. Structure of substrate for IRED activity a) 2‐methyl‐1‐pyrroline; and RedAm activity with b) cyclohexanone and c) 2‐hexanone and methylamine (MeNH_2_) as amine donor. d) Heat map showing initial rates (μmol/mLmin) calculated in presence of NADPH and NADH (as cofactors) and at pH 7 and 9 (TRIS−HCl 50 mM) at 30 °C. All experiments were conducted in duplicates.

Recombinant IREDs were placed in a 96‐well plate format (in duplicate), and enzymes were expressed. Most IREDs showed good expression levels in the soluble fraction (see SDS‐PAGE analysis in Supporting Information). IRED activities were screened (as clarified lysates) against model compound substrates: 2‐methyl‐1‐pyrroline to test imine reductase activity (IRED activity, Scheme 2a) and cyclohexanone or 2‐hexanone with methylamine as amino donor (1 : 8 eq.) to test reductive aminase activity (RedAm activity, Scheme 2b and 2c); all at pH 7 and 9 and in the presence of either NADPH or NADH.

A summary of the results is shown in Scheme 2d as a heat map based on the comparison of initial rates calculated following cofactor consumption, using empty plasmid‐clarified lysate and free‐clarified lysate reaction as controls. IREDs belonging to the D187−G196 subgroup showed modest to good expression levels; remarkably, pQR2598, 2600 and 2611 were highly active showing a broad range of activity with all substrates, cofactors and at both pH values; however, pQR2604, 2608 and 2618 showed poor activity levels for all substrates and conditions; therefore, we can infer that IREDs broad activity cannot be associated to these particular amino acid residue subgroups. IRED pQR2602 (D187−A196 sub‐group) showed also similar broad activity. Only three IREDs (pQR2603, 2608 and 2621) showed extremely poor or no activity, which might be due to low expression levels or lack of appropriate substrates. The remaining IREDs expressed well but showed variable activity under the experimental conditions. Hence, based on our screening results, and in agreement with previous publications, IRED activity‐specificity is highly substrate and cofactor dependent, and cannot easily be associated to a specific subgroup or sequence motif.

Most IREDs described to date have a natural preference for NADPH;^[8]^ here, IRED activity for most of the selected enzymes showed also a preference for NADPH at pH 7. RedAm activity is usually observed in some IREDs, which is favoured in the presence of an excess of the amine donor (8 eq. in this work) and at pH 9. Here, RedAm activity with cyclohexanone was higher in the presence of NADPH; and on average, greater at pH 9 for most of the IREDs evaluated. In addition, “true” RedAms, an IREDs subset, can catalyse the reductive amination of prochiral ketones with equimolar concentrations of the amine donor and at pH 7;[^10^, ^21^] although these conditions were not tested in this study, from the phylogenetic perspective, pQR2598, 2600 and 2608 are more closely related to (*R*)‐IRED_Aory (first “true” RedAm described);^[10]^ however, based on our data, it is unclear if these IREDs are “true” RedAms, and this will be explored in the future.

IRED‐catalysed reactions with NADH as the cofactor would be advantageous as this cofactor is more stable and cheaper than NADPH, and it is also easier to regenerate enzymatically for applications at a large scale.^[13]^ Three IREDs (pQR2600, 2601 and 2602; all from different subgroups) showed good IRED and RedAm activities, able to almost equally accept NADH. In this respect, Borlinghaus et al. reported an alteration of cofactor specificity for (*R*)‐IRED_*Ms* (*Mixococcus stipitatus*) achieving a 2900‐fold improved NADH/NADPH specificity and enhanced activity for the reduction of 2‐methyl‐1‐pyrroline, concluding that the key amino acid residues for NADH specificity are R50Y (R33Y in *Ms*) and particularly K54R (K37R in *Ms*).^[36]^

All IREDs in this study have a conserved R50 (used as a selection criteria), whilst only 13 and 16 IREDs contained K54 and R54, respectively (Figure 1). Remarkably, and in contrast to previous findings, all the N187−A196 and E187−P196 IREDs subgroups have K54 (associated with low NADH specificity) but showed moderate to good activity with NADH as a cofactor for the reduction reactions (except pQR2606 with good activities in presence of NADPH). From this, we infer that E187 and N187 IREDs prefer NADH as cofactor; this may be associated not only with residue 187 but also residues near the cofactor binding site motif (position 54) and active site motif (position 196).

In addition, reductive aminase activity has mainly been tested towards aromatic ketones with NADPH as the cofactor and methylamine as the amine.^[37]^ Our results show that most IREDs had RedAm activity towards 2‐cyclohexanone mainly in presence of NADPH; however, and irrespective of the level of activity, RedAm activity towards the aliphatic ketone 2‐hexanone was higher mainly in presence of NADH compared to NADPH and on average the best activity was observed at pH 7 (Scheme 2d).

### IREDs catalysed the synthesis of THIQs

**Scheme 3:**
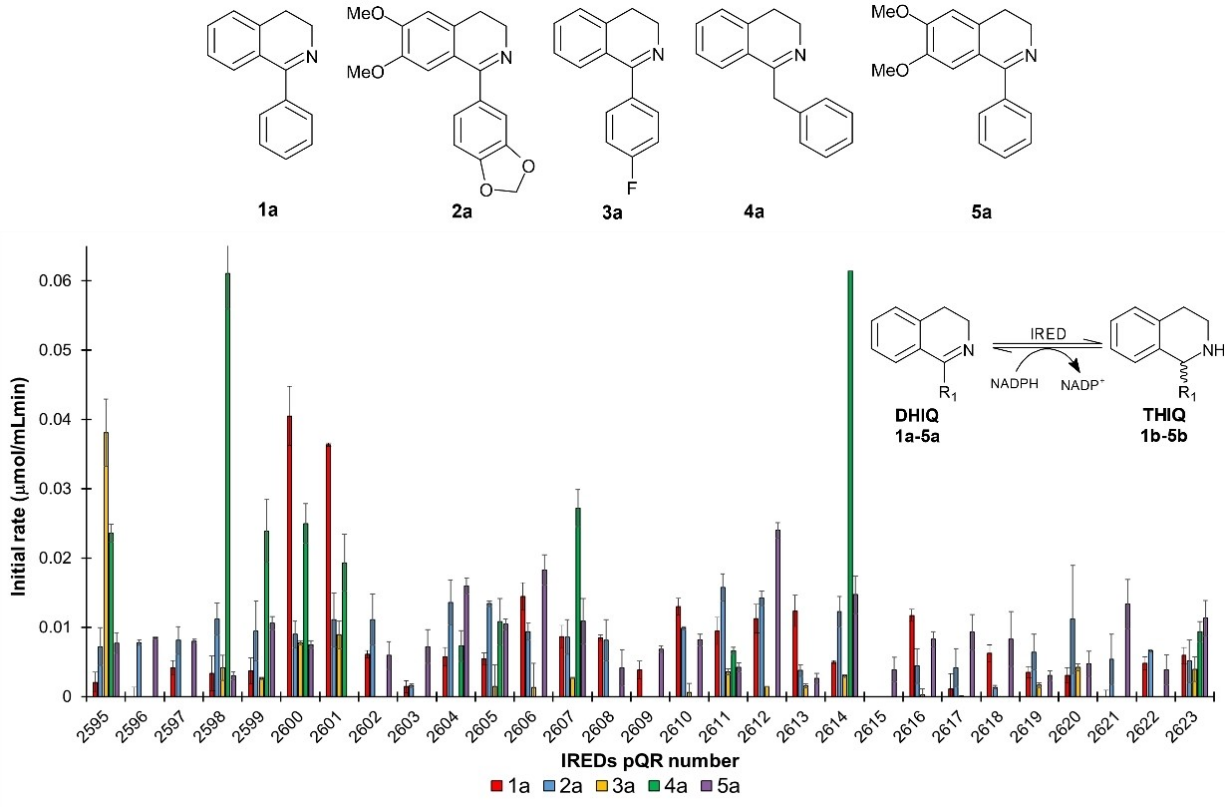
IREDs activity towards DHIQs **1 a**–**5 a** for the synthesis of THIQs **1 b**–**5 b**. Initial rates were calculated in duplicates after up to 2 h reaction: 20 μL of clarified lysate was mixed with 180 μL of reaction mixture containing **1 a**–**5 a** (1 mM), DMSO (2.5 % v/v) and NADPH (0.2 mM) in TRIS−HCl buffer (50 mM, pH 7) at 30 °C.

THIQs are important scaffolds for the synthesis of active pharmaceutical ingredients such as 1‐methyl−THIQ, salsolidine and solifenacin.^[29]^ Here, the reduction of five aryl−DHIQs (**1 a**–**5 a**, Scheme 3) into their respective THIQs (**1 b**–**5 b**) in the presence of NADPH (1.2 eq.) was studied. IRED activity results are shown in Scheme 3 as initial rates. It has previously been reported that W195 plays an important role in DHIQ substrate accommodation potentially causing steric hindrance and as an explanation for the poor activity of IREDs, and this residue is particularly critical for substrate binding in (*S*)‐IREDs.^[30]^ It has also been shown that F194 is important for substrate accommodation for 6,7‐dimethoxylated‐aryl−DHIQs (**2 a** and **5 a** in this study); hence, a double mutant F194M−W195F showed up to 6.8‐fold improvement in catalytic activity for this substrate type.^[38]^

In our study, the D187 IREDs associated to (*R*)‐IRED_Aory have a consensus M194 and Y/S/T/N195 residues; other D187 IREDs have low conservation for the 194 residue with either M/F/C194, and highly conserved residue W195; these IREDs subgroup were the most active towards the DHIQs evaluated. The Y187 IREDs had a highly conserved F194−W/L195 showing overall moderate activity, except pQR2599 and 2607, where both were highly active towards **4 a**. The E187 and N187 IREDs contained highly conserved L194−L195 and M194−Y/W195, respectively; both subgroups exhibited low activity towards the DHIQs, this might be associated to these specific amino acid residues and their preference for NADH to catalyse the imine reduction.

In this work, four IREDs (pQR2595, 2600, 2601 and 2612) were selected for the reduction of **1 a**, **3 a**, **4 a** and **5 a** into their respective THIQs (Table 2). These IREDs belong to the D187 subgroup with a predicted (*R*)‐stereoselectivity based on sequence analysis. Activity towards **2 a** was inconclusive as most IREDs showed similar activity values (at around 0.01 μmol/mLmin), and therefore this compound was not used further. THIQ **4 b** was difficult to synthesise chemically due to its high instability, hence reaction yields were calculated based on **4 a** consumption. Most THIQ reaction yields after 24 h were relatively low (21–44 %) except for **4 b** which was catalysed by pQR2600 (74 %). Interestingly, pQR2600 and 2601 accepted **1 a** but showed very low activity towards **3 a**, which only differs by the presence of a fluorine in the *para* position of the phenyl group in **1 a**; conversely, pQR2595 exhibited high activity towards **3 a** and poor activity towards **1 a**.

**Table 2:** Synthesis of THIQs catalysed by selected novel IREDs and coupled reaction IREDs with glucose‐6‐phosphate dehydrogenase (G6PDH). All experiments were conducted in duplicate.

IRED pQR	DHIQ Substrate	THIQ yields [%]		Product *ee*
		IRED	IRED+G6PDH	
2595	**3 a**	33	74	(*R*)‐**3 b** >99 % *ee*
	**4 a**	44^a^	90^a^	ND
2600	**1 a**	21	84	(*R*)‐**1 b** 93 % *ee*
	**4 a**	74^a^	92^a^	ND
2601	**1 a**	23	98	(*R*)‐**1 b** >99 % *ee*
	**4 a**	21^a^	91^a^	ND
2612	**5 a**	23	76	(*S*)‐**5 b** >99 % *ee*

^a^ Based on the conversion of **4 a**. ND, not determined.

**Scheme 4:**
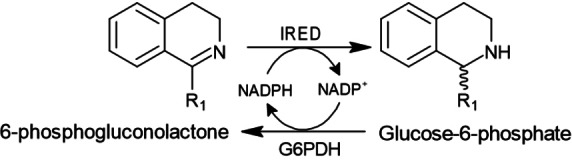
Synthesis of THIQs catalysed by IREDs coupled with glucose‐6‐phosphate dehydrogenase (G6PDH) for cofactor recycling.

NADPH cofactor recycling is a strategy widely used to increase reaction yields and reduce process cost. *In‐situ* NADPH recycling was carried out by coupling IREDs reaction to a G6PDH with glucose‐6‐phosphate as the co‐substrate^[39]^ (Scheme 4). As expected, this allowed us to significantly increase THIQ reaction yields by 1.2‐ to 4.4‐fold, reaching for example 98 % of **1 b**, catalysed by pQR2601 (Table 2).

Notably, pQR2595 and 2601 were highly stereoselective with >99 % (*R*)‐**3 b** and (*R*)‐**1 b**, respectively. Likewise, (*R*)‐**1 b** with a 93 % *ee* was obtained with pQR2600. For pQR2612, the main product was (*S*)‐**5 b** (>99 % *ee*) in contrast to its predicted stereoselectivity based on residue D187 (Table 2). IRED inverse stereoselectivity has also been reported previously, although this is not fully understood yet and it has been reported that P139 and F194 are potentially better predictors of stereoselectivity other than only D/Y187.^[17]^ Nevertheless, pQR2612 (with moderate activity towards model substrates) contains a P139 and an unusual C194 (the sole member of our IRED panel with a Cys in this position) (Figure 1). Several IREDs reported for the reduction of aryl−DHIQs into their respective aryl−THIQs are (*S*)‐selective, which have been described to be more tolerant towards bulkier substrates.^[22]^ Some (*R*)‐selective IREDs able to reduce aryl−DHIQs have been also reported but their substrate scope is more limited.^[30]^

## Conclusions

IREDs are promising biocatalysts for the synthesis of primary, secondary, and tertiary chiral amines. Based on the phylogenetic analysis of the identified IREDs, they were grouped into four subgroups based on amino acid residue 187, with Asp, Tyr, Glu or Asn in this key position. Here, in agreement with previously reported work, IRED sequence‐activity relationships are highly substrate dependent, and activity cannot be limited to any subgroup or sequence motif. Most of the twenty‐nine novel IREDs identified here expressed well. The majority showed one or more type of activity (IRED and RedAm), with a preference for NADPH as the cofactor. However, three IREDs were able to accept both NADPH and NADH. Remarkably, E/N187 IRED subgroups showed good activity and preference for NADH, which is advantageous from a process and cost perspective. In addition, reductive aminase activity towards 2‐hexanone was favoured mainly in the presence of NADH, which needs to be investigated further. Four selected IREDs were also applied in the synthesis of relevant THIQs, where coupling this reaction to a G6PDH cofactor recycling system significantly improved reaction yields, of up to 98 %. Although the stereoselectivity of IREDs is not fully understood yet; here, three selected IREDs matched the predicted stereoselectivity based on sequence analysis; however, one IRED showed inverse stereoselectivity which cannot be fully explained at this stage.

## Material and methods

### Chemicals, strains, and plasmids

All chemicals were purchased from Merck KGaA (Sigma‐Aldrich) and molecular biology reagents were from New England Biotechnology, otherwise stated. Strains used in this study were: *Streptomyces ambofaciens* DSM 40053*, Streptomyces coelicolor* 2612*, Streptomyces curacoi* DSM 40107*, Streptomyces peuceticus* ATCC 27952*, Streptomyces rimosus* DSM 41429*, Streptomyces mobaraensis* DSM 40847*, Saccharopolyspora erythraea* DSM 40517, *Streptoalloteichus hindustanus* DSM 44523 *and Streptosporangium roseum* DSM 43021.

### IREDs genome mining, cloning and expression

A sequence‐based genome mining strategy was used to identify IREDs in selected strains in BLASTp with IRED protein sequence from *S. kanamyceticus* (Uniprot Q1EQE0) as the model IRED. Then, a motif‐based analysis confirmed enzyme class with Jalview 2.11.5 bioinformatics software. The phylogenetic tree was produced using Clustal Omega, dendrogram and further editing was performed in iToL online tool. IREDs cloning was carried out following a one‐pot restriction‐ligation reaction method with either SapI or BsaI and the modified pET28a+ and pET29a+ vectors, respectively.^[35]^ Cloning reactions were transformed into chemically competent *E. coli* NovaBlue (propagation strain); then, confirmed plasmids containing genes of interest were transformed into chemically competent *E. coli* BL21(DE3) or *E. coli* Rosetta 2(DE3) (expression strain). All recombinant IREDs have a C‐terminal His_6_Tag.

### Enzymes expression and cell lysis

Twenty‐nine IREDs were selected (pQR2595‐2623) for further expression and activity screening experiments. They were put in a 96‐deep‐well plate (position from A1 to C5) in duplicate (position from D1 to F5), including *E. coli* BL21(DE3) with empty pET29a+ as negative control (C6 and F6). Expression was with 1 mL of TB‐broth per well with kanamycin 50 μg/mL at 37 °C and 1200 rpm in a Thermomixer™ C (Eppendorf, UK), IPTG induction (0.5 mM final concentration) to an OD_600nm_ ∼1.5 and the temperature was then reduced to 25 °C for 15 h. The plate containing IREDs was then centrifuged (4500 rpm at 4 °C for 30 min) and cell pellets were resuspended in 0.2 mL of 50 mM TRIS−HCl buffer pH 7. Cells were disrupted by partially submerging the IRED‐plate in a water bath sonicator (Bransonic 2800 CPX2800H‐E, Branson) as previously described.^[40]^ The clarified lysates were recovered in a fresh 96‐well plate and kept at 4 °C for enzymatic activity screening and protein quantification.

### Activity screening assay

Enzymatic kinetic assays were performed for substrate screening, using 2‐methyl‐1‐pyrroline (10 mM) for IRED activity, and cyclohexanone or 2‐hexanone (20 mM) with methylamine (160 mM) as amine donor for RedAm activity. All reactions were performed mixing 20 μL of clarified lysate with 180 μL of reaction mixture containing substrates and either NADPH or NADH (0.2 mM) in TRIS−HCl buffer (50 mM) adjusted to pH 7 or 9 and incubated at 30 °C. NADPH/NADH depletion was monitored at 340 nm for up to 30 min in a plate reader (CLARIOstar Plus, BMG Labtech) and the initial rates (μmol/mLmin) were then calculated by using ϵNAD(P)H 340 nm=6220 M^−1^cm^−1^. Additional control reactions were performed following the above procedure using empty plasmid‐clarified lysate and free‐clarified lysate reaction as controls. IREDs activity was calculated by subtracting their activity values minus activity of controls. All screening reactions were performed in duplicate.

### THIQs enzyme screening and synthesis

Initial IRED activity for THIQs synthesis was performed as described in the Activity screening assay section. DHIQs and THIQs were all chemically synthesised in our Lab (except DHIQ‐**1 b** which was purchased from Fluorochem); their synthesis and characterization is shown in the Supporting Information. For the reactions, 20 μL of clarified lysate was mixed with 180 μL of reaction mixture containing DHIQ **1 a**–**5 a** (1 mM), DMSO (2.5 % v/v) and NADPH (0.2 mM) in TRIS−HCl buffer (50 mM, pH 7) and incubated in the plate reader for up to 2 h at 30 °C. Then initial rates were calculated using control reactions as explained above. Small‐scale THIQ syntheses (1 mL) were performed with selected IREDs and respective DHIQs. Reaction mixtures contained IRED clarified lysate (20 % v/v), DHIQ (2.5 mM), DMSO (2.5 % v/v) and NADPH (3 mM, 1.2 eq.) in TRIS−HCl buffer (50 mM, pH 7) and were incubated at 30 °C for up to 24 h in Thermomixer™ C.

In order to improve reaction yields and reduce cofactor utilisation, IRED reactions were coupled with G6PDH.^[39]^ One mL of reaction mixture contained selected IRED and G6PDH clarified lysates (20 % v/v of each), DHIQ (2.5 mM), DMSO (2.5 % v/v), NADP^+^ (0.5 mM), glucose‐6‐phosphate (20 mM) in TRIS−HCl buffer (50 mM, pH 7) and incubated at 30 °C for up to 24 h in Thermomixer™ C.

All bioconversion reactions were conducted in duplicate. Reactions were quenched by adding 1 volume of 0.5 % TFA (v/v in milliQ water), samples were centrifuged (10000 rpm at 4 °C for 15 min) and the supernatants were analysed by HPLC.

### DHIQ and THIQ analyses

The amounts of DHIQs and THIQs were quantified by HPLC with an Ultimate 3000+ UHPLC (ThermoFisher Scientific) fitted with an ACE−C18 column and 0.1 % TFA (v/v in milliQ water) and acetonitrile (CH_3_CN) as mobile phases. Gradient elution was performed from 30 to 50 % CH_3_CN for 8 min, followed by 100 % CH_3_CN for 2 min and 30 % CH_3_CN for 2 min, at a constant flow rate 0.6 mL/min and column oven at 30 °C. Compounds were detected at 260 nm, and retention times for **1 a**, **3 a**, **4 a** and **5 a** were 3.3, 3.6, 3.9 and 5.6 min respectively; and for **1 b**, **3 b**, and **5 b** were 4.1, 4.5 and 5.2 min respectively (see chromatograms in Supporting Information). All quantitative analyses were performed measuring peak area using the external standard method. The enantiomeric excess (*ee*%) was determined with a Chiralpak® AD−H column, with 98.5 : 1.5:0.1 n‐hexane:EtOH:diethylamine as the mobile phase, at a flow rate 1 mL/min for 20 min and detection at 220 nm (see chromatograms in Supporting Information).

## Conflict of interest

None declared.

1

## Supporting information

As a service to our authors and readers, this journal provides supporting information supplied by the authors. Such materials are peer reviewed and may be re‐organized for online delivery, but are not copy‐edited or typeset. Technical support issues arising from supporting information (other than missing files) should be addressed to the authors.

Supporting Information

## Data Availability

The data that support the findings of this study are available in the supplementary material of this article.
