# Supplementary material for: The Discovery of Imine Reductases and their Utilisation for the Synthesis of Tetrahydroisoquinolines
Source: ChemCatChem. 2023 Jan 11;15(3):e202201126. doi: 10.1002/cctc.202201126 (PMC10107726; doi:10.1002/cctc.202201126)
Supplement: Supplementary file 1 — Supporting Information [file CCTC-15-0-s001.pdf]

ChemCatChem

Supporting Information

The Discovery of Imine Reductases and their Utilisation for the Synthesis of Tetrahydroisoquinolines

Max Cárdenas-Fernández,* Rebecca Roddan, Eve M. Carter, Helen C. Hailes, and John M. Ward*

Supporting Table 1: IREDs panel and pQR numbers (the Ward group plasmid identifier). Expression host system either *E. coli* BL21(DE3) or *E. coli* Rosetta 2(DE3)*

pQR number	Microorganism source	Protein accession number	Protein length (n°. amino acids)	Protein molecular mass (kDa)
2595	*Streptomyces ambofaciens*	WP_053126085.1	294	31
2596*		WP_079029964.1	296	30.6
2597		WP_053126081.1	305	32
2598	*Streptomyces coelicolor*	NP_626724.1	311	32.1
2599	*Streptomyces peuceticus*	ATW51439.1	302	31.5
2600		ATW48861.1	293	30.3
2601	*Streptomyces rimosus*	GCD40644.1	293	30.5
2602		GCD41322.1	294	30
2603*		GCD43400.1	317	33.5
2604		GCD43831.1	310	31.1
2605		GCD48278.1	301	31
2606	*Saccharopolyspora erythraea*	PFG99428.1	300	30.9
2607		PFG98216.1	291	30.5
2608*		PFG94490.1	293	30.6
2609*	*Streptomyces curacoi*	WP_062153271.1	297	31.1
2610	*Streptomyces mobaraensis*	EMF00717.1	294	31
2611		EME99395.1	290	30.1
2612*		EMF02685.1	301	30.6
2613		EMF01349.1	300	31.6
2614	*Streptoalloteichus hindustanus*	WP_073480793.1	300	30.7
2615*		WP_073483347.1	295	30.6

2616		WP_073486480.1	296	31
2617*		WP_073479465.1	289	30.4
2618		WP_073484516.1	292	29.4
2619*	*Streptosporangium roseum*	ACZ87755.1	302	32
2620		ACZ88126.1	295	30.9
2621		ACZ88887.1	288	29.2
2622*		ACZ87862.1	293	30.3
2623*		ACZ86980.1	287	30.4

Supporting Figure 1. SDS-PAGE analysis of IREDs in 1 mL Terrific broth containing kanamycin 50 µg/mL per well at 1200 rpm at 37 °C until OD600 nm ~1.5; then induced with IPTG 0.5 mM and temperature reduced to 25 °C for additional 15 h. This figure corresponds to the analysis of the soluble fraction (clarified lysate), after cell lysis by sonication run in different experiments (red boxes). PageRuler™ Prestained Protein Ladder, 10 to 180 kDa (Thermo Scientific™)

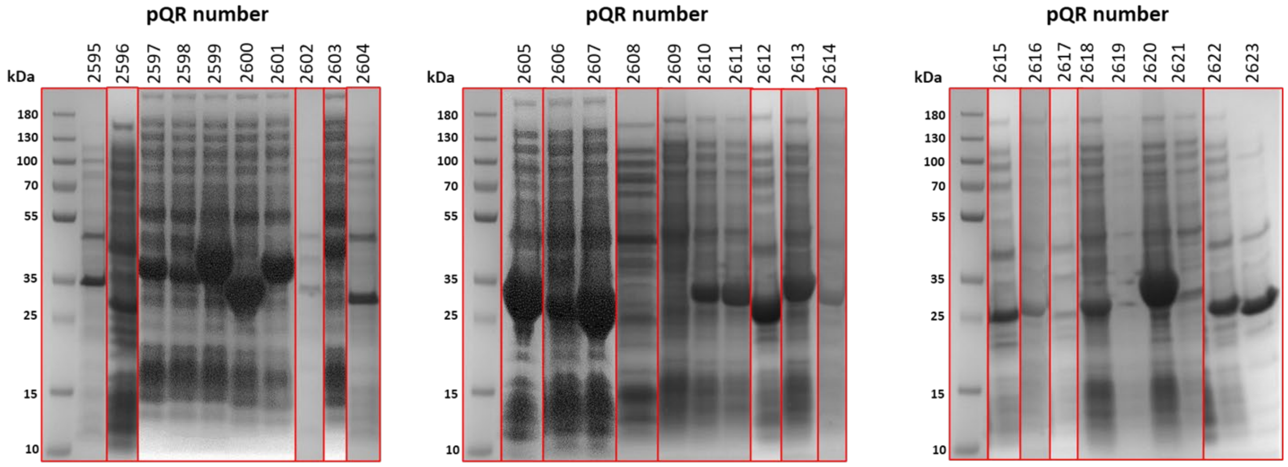

Supporting Figure 2. HPLC chromatograms obtained using an Ultimate 3000+ UHPLC (Thermofisher Scientific) fitted with an ACE-C18 column and 0.1% TFA (v/v in milliQ water) and acetonitrile (CH_3CN) as mobile phases. Gradient elution was performed from 30 to 50% CH_3CN for 8 min, followed by 100% CH_3CN for 2 min and 30% CH_3CN for 2 min, at a constant flow rate of 0.6 mL/min and column oven at 30 °C. Compounds were detected at 260 nm. A) DHIQ-**1a** and THIQ-**1b**; B) DHIQ-**3a** and THIQ-**3b**; C) DHIQ-**4a** and THIQ-**4b**; and D) DHIQ-**5a** and THIQ-**5b**.

A)

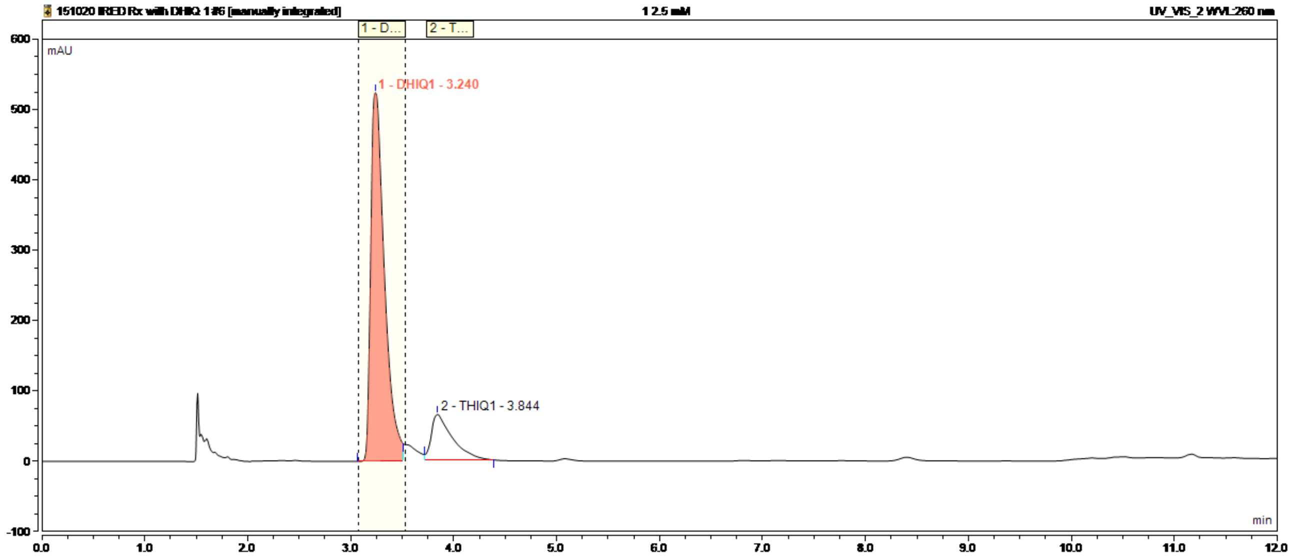

B)

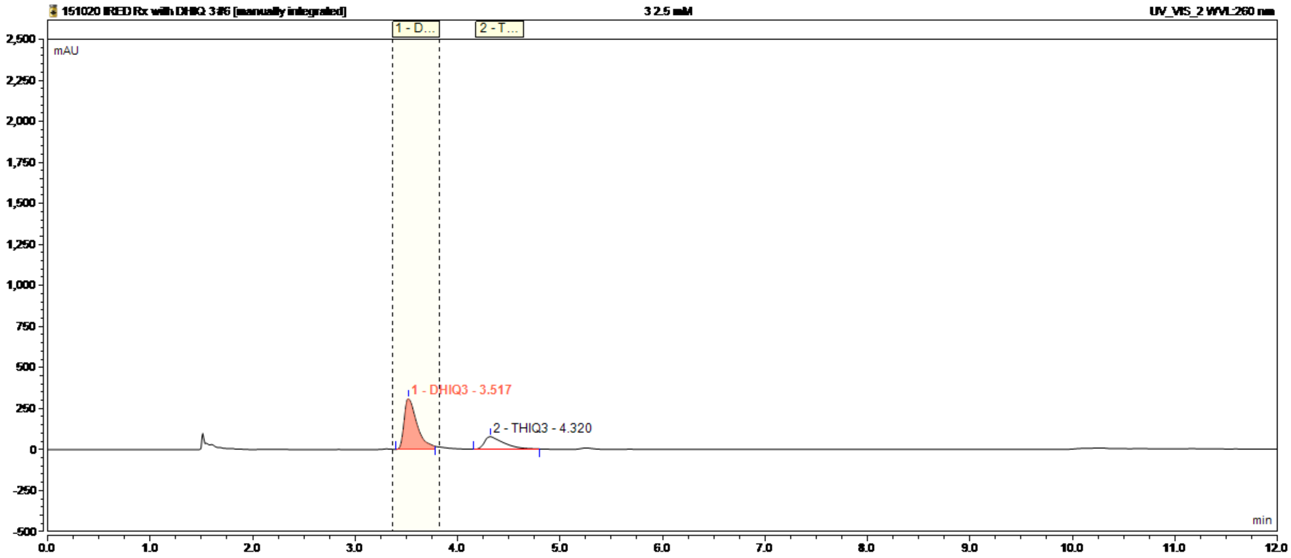

C)

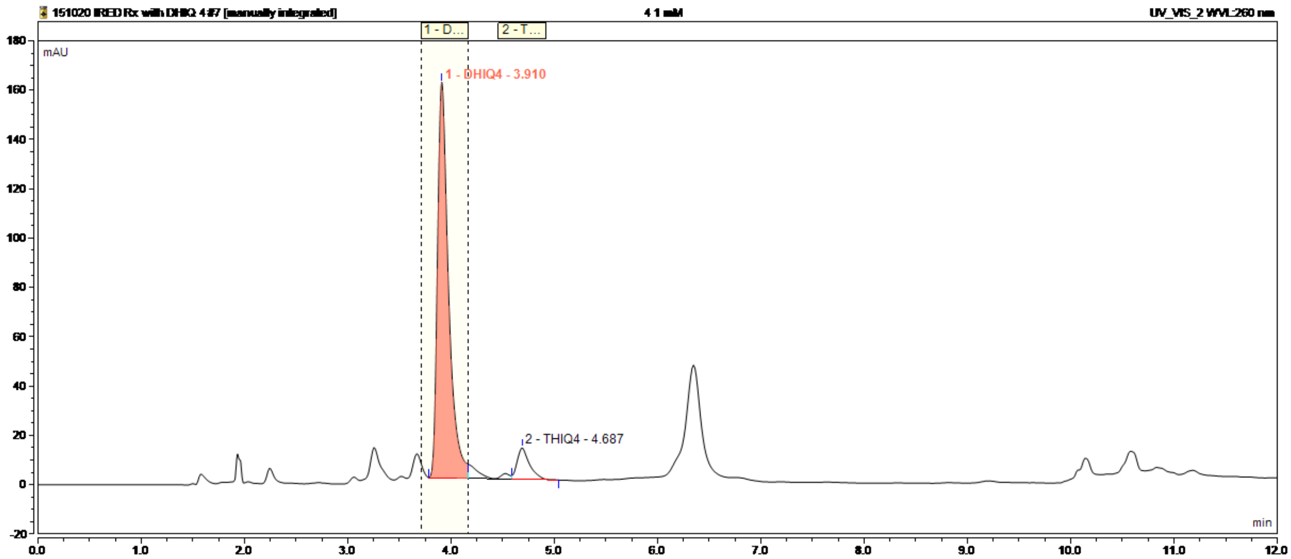
151020 IRED Rx with DHIQ 4 #7 [manually integrated]
4 1 mM
UV_VIS_2 WVL:260 nm
1 - D...
2 - T...
mAU
1 - DHIQ4 - 3.910
2 - THIQ4 - 4.687
min
180
160
140
120
100
80
60
40
20
0
-20
0.0
1.0
2.0
3.0
4.0
5.0
6.0
7.0
8.0
9.0
10.0
11.0
12.0

D)

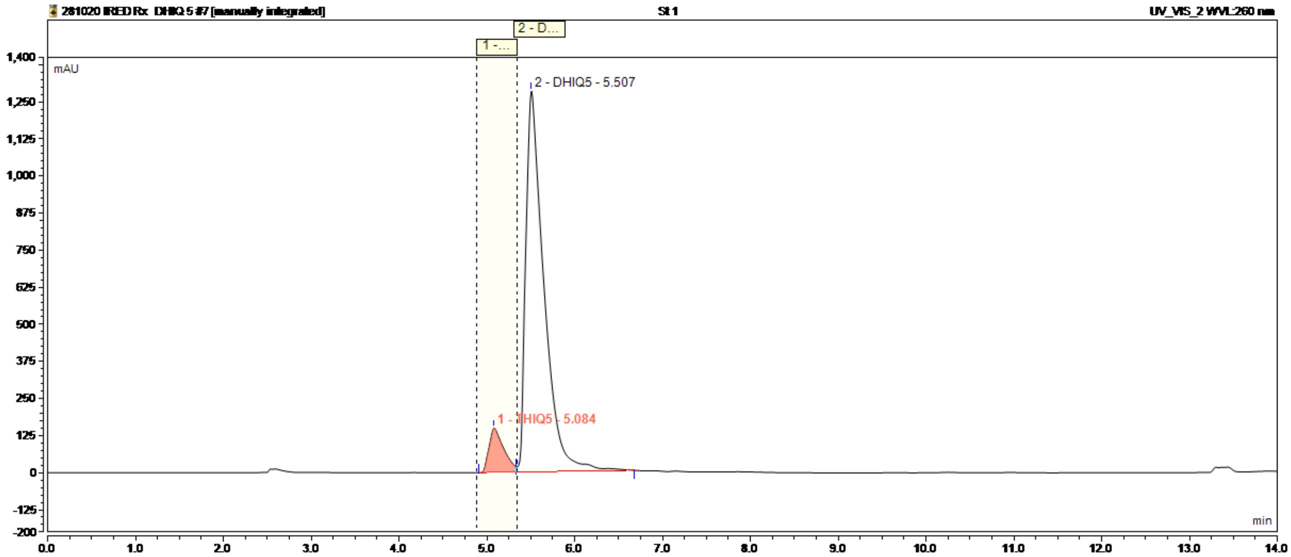
281020 IRED Rx DHIQ 5 #7 [manually integrated]
St 1
UV_VIS_2 WVL:260 nm
2 - D...
1 -...
mAU
2 - DHIQ5 - 5.507
1 - THIQ5 - 5.084
min
1,400
1,250
1,125
1,000
875
750
625
500
375
250
125
0
-125
-200
0.0
1.0
2.0
3.0
4.0
5.0
6.0
7.0
8.0
9.0
10.0
11.0
12.0
13.0
14.0

Supporting Figure 3. Chiral HPLC analysis was used to determine the enantiomeric excess (*ee*%) with an Chiralpak® AD-H column, with 98.5:1.5:0.1 n-hexane:EtOH:diethylamine as the mobile phase, at a flow rate 1 mL/min for 20 min and detection at 220 nm. A) THIQ-**1b**; B) THIQ-**3b**; and C) THIQ-**5b**.

A) Dark green, DHIQ-**1a** (t_R = 8 min); Red, (1*S*)-THIQ-**1b** (t_R = 11 min) and (1*R*)-THIQ-**1b** (t_R = 15 min). Reactions catalysed by: Blue, pQR2600 and Light green, pQR2601.

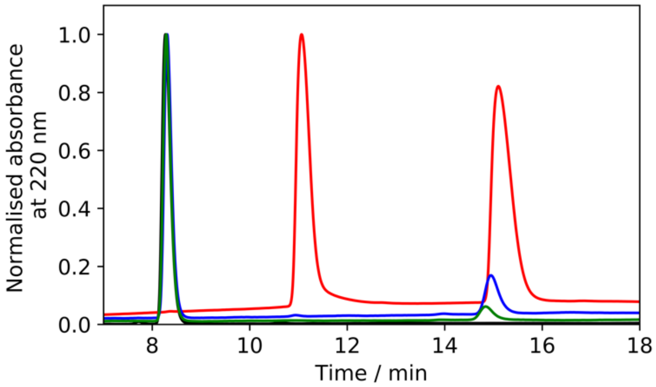

B) Red, (1*S*)-THIQ-**3b** (t_R = 12-13 min) and (1*R*)-THIQ-**3b** (t_R = 15-16 min). Reactions catalysed by: Blue, pQR2595; Orange, pQR2598; Green, pQR2600; Red, pQR2601; and Purple, pQR2614.

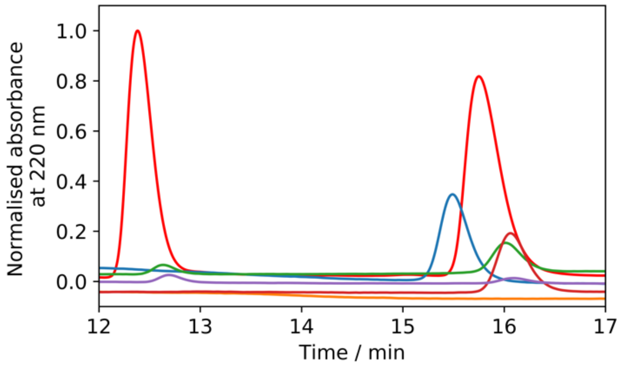

C) Blue, (1*S*)-THIQ-**5b** (t_R = 9 min) and (1*R*)-THIQ-**5b** (t_R = 13 min). Reaction catalysed by: Orange, pQR2612.

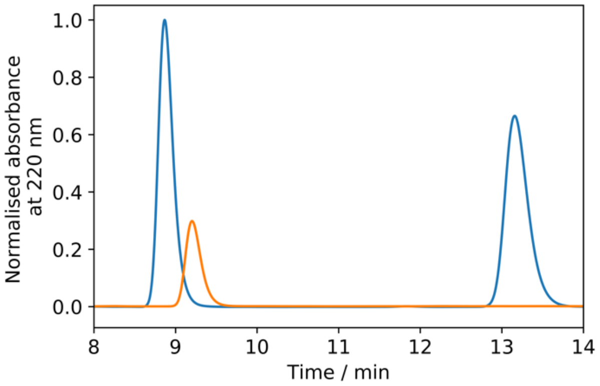

Small molecule syntheses and characterisation

Chemical Reagents

Geduran® Si 60 silica (43-60 μM) was used for silica column chromatography. Thin layer chromatography analysis was performed using plates with a silica gel matrix on an aluminium support and the plates were visualised using ultraviolet light (254 nm). All reagents were obtained from commercial sources unless otherwise specified.

Chemical Characterization

^{1}H and ^{13}C NMR spectra were obtained using Bruker Advance III 600 MHz and 700 MHz spectrometers (as specified in the characterization data). Chemical shifts specified are relative to trimethylsilane (set at 0 ppm) and referenced to the residual, protonated NMR solvent. Coupling constants in ^{1}H-NMR spectra (*J*) are given in Hertz (Hz) and described as singlet (s), doublet (d), triplet (t), quartet (q), multiplet (m). Mass spectrometry data was obtained using a Waters Aquity UPLC-MS system (MS [ES+]).

N-Phenethylbenzamide[1]

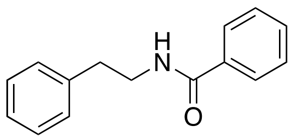

Under anhydrous conditions, a solution of phenethylamine (630 μL, 5.0 mmol, 1 e.q.) in dichloromethane (10 mL) was prepared and stirred at rt. Triethylamine (1.04 mL, 7.5 mmol, 1.5 e.q.) was added and the solution cooled to 0 °C. Benzoyl chloride (580 μL, 5.0 mmol, 1 e.q.) was added dropwise and the reaction stirred at 0 °C for 30 min. The reaction was warmed to room temperature and stirred for 30 min. The solution was concentrated under reduced pressure, resuspended in ethyl acetate (20 mL) and the organic phases washed with 1 M HCl (1 x 20 mL) and brine (1 x 20 mL). The organic phase was dried with anhydrous sodium sulfate and concentrated under reduced pressure. The resulting residue was purified by column chromatography (10 – 50% ethyl acetate in *n*-hexane) to give the product as a white solid (1.1 g, 96%). ^{1}H-NMR (700 MHz, DMSO-d$_6$) δ 8.55 (1H, t, *J* = 5.3 Hz, N*H*), 7.80 (2H, m, Ar*H*), 7.50 (1H, m, Ar*H*), 7.44 (2H, t, *J* = 7.9 Hz, Ar*H*), 7.28 (2H, t, *J* = 7.5 Hz, Ar*H*), 7.23 (2H, m, Ar*H*), 7.19 (1H, t, *J* = 7.2 Hz, Ar*H*), 3.47 (2H, app. q, *J* = 7.5 Hz, C*H*$_2$NH), 2.84 (2H, t,

J = 7.5 Hz, CH_2CH$_2$NH); ^{13}C-NMR (176 MHz, DMSO-d$_6$) δ 166.2, 139.6, 134.6, 131.1, 128.7, 128.4, 128.3, 127.1, 126.1, 40.9, 35.1; MS m/z [ES$^+$] 226 ([M+H]$^+$, 100%).

1-Phenyl-3,4-dihydroisoquinoline[2] 1a

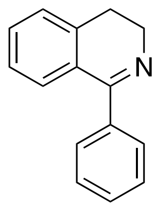

A mixture of *N*-phenethylbenzamide (500 mg, 2.20 mmol, 1 e.q.) and polyphosphoric acid (3.25 g, 33.2 mmol, 15 e.q.) was stirred at 200 °C for 6 h, then rt for 18 h. Water (20 mL, 0 °C) was added and the mixture sonicated until a homogeneous solution was obtained. The pH of the solution was adjusted to 7 by addition of 2 M NaOH, then extracted into diethyl ether (3 x 20 mL). The organic phases were dried with anhydrous sodium sulfate and concentrated under reduced pressure to give the pure product as a brown oil (413 mg, 90%). ^{1}H-NMR (700 MHz, CDCl$_3$,) δ 7.62 – 7.59 (2H, m, ArH), 7.46 – 7.41 (3H, m, ArH), 7.39 (1H, td, J = 7.2, 1.7 Hz, ArH), 7.29 – 7.23 (3H, m, ArH), 3.88 – 3.84 (2H, m, NCH_2), 2.82 (2H, t, J = 7.5 Hz, CH_2CH$_2$N); ^{13}C-NMR (176 MHz, CDCl$_3$) δ 167.5, 139.0, 139.0, 130.9, 129.5, 128.9, 128.9, 128.3, 128.1, 127.5, 126.7, 47.7, 26.4; MS m/z [ES$^+$] 208 ([M+H]$^+$, 100%).

***N*-(3,4-Dimethoxyphenethyl)benzo[*d*][1,3]dioxole-5-carboxamide[3]**

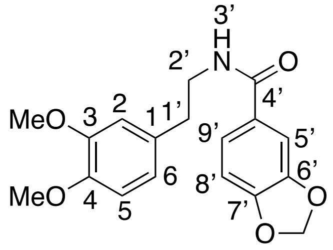

Under anhydrous conditions, a solution of 3,4-dimethoxyphenethylamine (339 μL, 2.00 mmol) in dichloromethane (8 mL) was prepared. Triethylamine (416 μL, 3.00 mmol) was added and the reaction mixture cooled to 0 °C. Piperonyloyl chloride (269 μL, 2.00 mmol) was added and the reaction mixture stirred at 0 °C for 30 min then rt for 1 h. The solution was concentrated under reduced pressure, resuspended in dichloromethane (20 mL) and washed with 1 M HCl (2 x 20 mL),

1 M NaOH (2 x 20 mL) and brine (2 x 20 mL). The organic phase was dried (anhydrous Na_2SO_4) and concentrated under reduced pressure. The residue was purified by column chromatography (3% MeOH in dichloromethane) to give *N*-(3,4-dimethoxyphenethyl)benzo[*d*][1,3]dioxole-5-carboxamide as a white solid (535 mg, 81%). ^{1}H-NMR (700 MHz, $CDCl_3$) δ 7.21 (1H, d, *J* = 1.7 Hz, 5'-H), 7.19 (1H, dd, *J* = 8.1, 1.7 Hz, 9'-H), 6.83 (1H, d, *J* = 8.1 Hz, 8'-H), 6.79 (1H, d, *J* = 8.1 Hz, 5-H), 6.76 (1H, dd, *J* = 8.1, 1.9 Hz, 6-H), 6.74 (1H, d, *J* = 1.9 Hz, 2-H), 6.01 (2H, s, OC*H*$_2$O), 6.00 – 5.95 (1H, m, 3'-H), 3.97 (3H, s, OC*H*$_3$), 3.85 (3H, s, OC*H*$_3$), 3.67 (2H, q, *J* = 6.8 Hz, 2'-H), 2.86 (2H, t, *J* = 6.8 Hz, 1'-H); ^{13}C-NMR (176 MHz, $CDCl_3$) δ 166.8, 150.4, 149.2, 148.1, 147.9, 131.5, 129.0, 121.4, 120.8, 112.1, 111.5, 108.1, 107.6, 101.8, 56.1, 56.0, 41.4, 25.4; MS *m/z* [ES$^+$] 330 ([M+H]$^+$, 100%).

1-(Benzo[*d*][1,3]dioxol-5-yl)-6,7-dimethoxy-3,4-dihydroisoquinoline[3] 2a

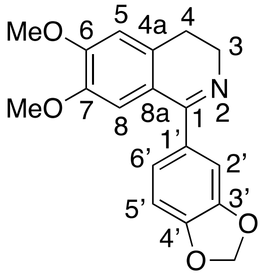

Under anhydrous conditions, a solution of *N*-(3,4-dimethoxyphenethyl)benzo[*d*][1,3]dioxole-5-carboxamide (530 mg, 1.61 mmol) in ethyl acetate (8 mL) was prepared and phosphorus oxychloride (299 μL, 3.22 mmol) was added dropwise over 5 min. The reaction was heated under reflux for 6 h then warmed to rt and quenched by the dropwise addition of water (1 mL) over 15 min. Dichloromethane (40 mL) was added and the organic phases washed with sat. aq. K_2CO_3 (3 x 20 mL). The aqueous layer was extracted with dichloromethane (3 x 20 mL) then the organic phases combined, dried (anhydrous Na_2SO_4) and concentrated under reduced pressure. The resulting residue was resuspended in dichloromethane (20 mL) and extracted into 2 M HCl (3 x 30 mL). The pH of the aqueous washings was adjusted to 13 and extracted with dichloromethane (3 x 30 mL). The organic phases were dried (anhydrous Na_2SO_4) and concentrated under reduced pressure to give 1-(benzo[*d*][1,3]dioxol-5-yl)-6,7-dimethoxy-3,4-dihydroisoquinoline as a yellow oil (340 mg, 67%). ^{1}H-NMR (700 MHz, $CDCl_3$) δ 7.14 (1H, d, *J* = 1.6 Hz, 2'-H), 7.11 (1H, dd, *J* = 7.9, 1.6 Hz, 6'-H), 6.87 – 6.84 (2H, m, 8-H and 5'-H), 6.78 (1H, s, 5-H), 6.02 (2H, s, OC*H*$_2$O), 3.95 (3H, s, OC*H*$_3$), 3.79 – 3.75 (5H, m, OC*H*$_3$ and 3-H), 2.72 (2H, t, *J* = 7.4 Hz, 4-H); ^{13}C-NMR (176 MHz, 176 MHz) δ 166.3,

151.2, 148.9, 147.8, 147.2, 133.0, 123.4, 121.5, 111.9, 110.4, 109.5, 108.0, 101.4, 56.4, 56.2, 53.6, 47.4, 26.2; MS *m/z* [ES$^+$] 312 ([M+H]$^+$, 100%).

1-(Benzo[*d*][1,3]dioxol-5-yl)-6,7-dimethoxy-1,2,3,4-tetrahydroisoquinoline[3] 2b

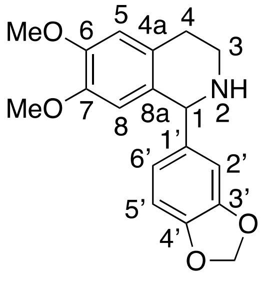

Under anhydrous conditions, a solution of 1-(Benzo[*d*][1,3]dioxol-5-yl)-6,7-dimethoxy-3,4-dihydroisoquinoline (43 mg, 0.14 mmol) in methanol (5 mL) was prepared and stirred. $NaBH_4$ (11 mg, 0.28 mmol) was added portion wise and the reaction stirred at rt for 2 h. The reaction was quenched by dropwise addition of water (10 mL). The pH was adjusted to 10 and the mixture was extracted with ethyl acetate (3 x 20 mL). The organic phases were combined, dried with anhydrous sodium sulfate and concentrated under reduced pressure to give the product as a pale yellow solid (39 mg, 86%). ^{1}H-NMR (700 MHz, $CDCl_3$) δ 7.76 – 7.72 (2H, m), 6.71 – 6.70 (1H, m), 6.61 (1H, s, 5-H), 6.27 (1H, s, 8-H), 5.93 (2H, 2xd, *J* = 1.5 Hz, OC*H*$_2$O), 4.98 (1H, s, 1-H), 3.86 (3H, s, 6-OC*H*$_3$), 3.67 (3H, s, 7-OC*H*$_3$), 3.21 (1H, dt, *J* = 12.3, 5.0 Hz, 3-*H*H), 3.06 – 3.00 (1H, m, 3-H*H*), 2.95 – 2.88 (1H, m, 4-*H*H), 2.73 (1H, dt, *J* = 15.9, 5.0 Hz, 4-H*H*); ^{13}C-NMR (176 MHz, $CDCl_3$) δ 147.9, 147.8, 147.3, 147.0, 138.9, 129.8, 127.7, 122.4, 111.5, 111.0, 109.3, 108.0, 101.1, 61.2, 56.4, 56.2, 41.9, 29.3; MS *m/z* [ES$^+$] 314 ([M+H]$^+$, 100%).

4-Fluoro-*N*-phenethylbenzamide[4]

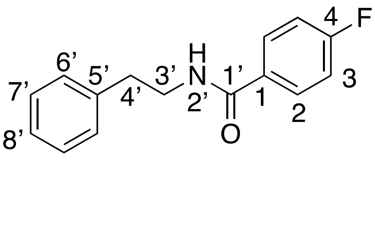

Under anhydrous conditions, a solution of phenethylamine (606 mg, 5.00 mmol) in dichloromethane (10 mL) was prepared and triethylamine (1.04 mL, 7.50 mmol) was added. The mixture was stirred and cooled to 0 °C. 4-Fluorobenzoyl chloride (592 mg, 5.00 mmol) was added and the reaction stirred at 0 °C for 30 min then rt for 2 h. The reaction was concentrated under reduced pressure,

resuspended in ethyl acetate (50 mL) and washed with 1 M HCl (3 x 50 mL), 1 M NaOH (3 x 50 mL) and brine (1 x 50 mL). The organic phase was dried with anhydrous sodium sulphate and concentrated under reduced pressure. The resulting residue was purified by column chromatography (20% ethyl acetate in *n*-hexane) to give the desired product as a white solid (1.08 g, 89%). ^{1}H-NMR (700 MHz, $CDCl_3$) δ 7.70 – 7.67 (2H, m, 2-H), 7.35 – 7.32 (2H, m, 7'-H), 7.27 – 7.23 (3H, m, 6'-H and 8'-H), 7.10 – 7.06 (2H, m, 3-H), 6.03 (1H, s, 2'-H), 3.72 (2H, q, J = 6.7 Hz, 3'-H), 2.94 (2H, t, J = 6.7 Hz, 4'-H); ^{13}C-NMR (176 MHz, $CDCl_3$) δ 166.6, 164.8 (d, $^{1}J_{CF}$ = 246 Hz), 138.9, 129.2, 129.2, 128.9, 128.9, 126.8, 115.7 (d, J = 22 Hz), 41.3, 35.8; MS m/z [ES$^+$] 244 ([M+H]$^+$, 100%).

1-(4-Fluorophenyl)-3,4-dihydroisoquinoline[4] 3a

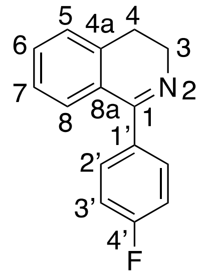

A mixture of 4-fluoro-*N*-phenethylbenzamide (500 mg, 2.06 mmol) and polyphosphoric acid (3.03 g, 30.9 mmol) was stirred at 200 °C for 6 h then cooled to rt. Water (0 °C, 20 mL) was added and the mixture sonicated to homogeneity. The pH was adjusted to 7 and the mixture extracted with diethyl ether (3 x 30 mL). The organic phases were combined, dried with anhydrous sodium sulfate and concentrated under reduced pressure to give the product as a pale brown oil (400 mg, 86%). ^{1}H-NMR (700 MHz, $CDCl_3$) δ 7.60 (2H, m, 2'-H), 7.40 (1H, td, J = 7.3, 1.7 Hz, 6-H or 7-H), 7.30 – 7.23 (3H, m, 5-H, 6-H or 7-H, and 8-H), 7.14 – 7.09 (2H, m, 3'-H), 3.86 – 3.82 (2H, m, 3-H), 2.83 – 2.79 (2H, m, 4-H); ^{13}C-NMR (176 MHz, $CDCl_3$) δ 166.5, 163.7 (d, $^{1}J_{CF}$ = 248 Hz), 139.1, 131.0, 130.9, 130.9, 128.7, 128.0, 127.7, 126.8, 115.3, (d, J = 22 Hz), 47.6, 26.4; MS m/z [ES$^+$] 226 ([M+H]$^+$, 100%).

1-(4-Fluorophenyl)-1,2,3,4-tetrahydroisoquinoline[4] 3b

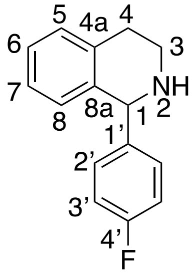

Under anhydrous conditions, a solution of 1-(4-fluorophenyl)-3,4-dihydroisoquinoline (66 mg, 0.29 mmol) in methanol (5 mL) was prepared and stirred. Sodium borohydride (22 mg, 0.59 mmol) was added portionwise and the reaction stirred at rt for 2 h. The reaction was quenched by the dropwise addition of water (10 mL). The pH was adjusted to 10 and the mixture was extracted with ethyl acetate (3 x 20 mL). The organic phases were combined, dried with anhydrous sodium sulfate and concentrated under reduced pressure to give the product as an orange oil (38 mg, 59%). ^{1}H-NMR (700 MHz, $CDCl_3$) δ 7.25 – 7.22 (2H, m, 2'-H), 7.16 – 7.14 (2H, m, 5-H and, 6-H or 7-H), 7.07 – 7.03 (1H, m, 6-H or 7-H), 7.02 – 6.98 (2H, m, 3'-H), 6.72 (1H, d, J = 8.0 Hz, 8-H), 5.10 (1H, s, 1-H), 3.26 (1H, dt, J = 11.9, 4.8 Hz, 3-*H*H), 3.12 – 3.08 (1H, m, 3-H*H*), 3.07 – 3.01 (1H, m, 4-*H*H), 2.83 (1H, dt, J = 16.0, 4.3 Hz, 4-H*H*); ^{13}C-NMR (176 MHz, $CDCl_3$) δ 162.2 (d, $^{1}J_{CF}$ = 247 Hz), 140.7, 138.2, 135.5, 130.7 (d, J = 8 Hz), 129.3, 128.1, 126.5, 125.8, 115.3 (d, J = 21 Hz), 61.5, 42.4, 29.8; MS *m/z* [ES^{+}] 228 ([M+H]$^{+}$, 100%).

***N*-Phenethyl-2-phenylacetamide[4]**

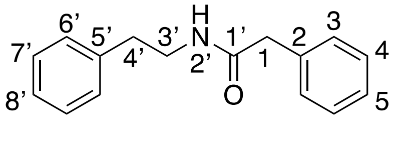

Under anhydrous conditions, a solution of phenethylamine (391 mg, 3.22 mmol) in dichloromethane (10 mL) was prepared. The solution was stirred, triethylamine (1.34 mL, 9.66 mmol) was added, and the reaction mixture was cooled to 0 °C. Phenylacetyl chloride (427 μL, 3.22 mmol) was added dropwise and the reaction warmed to rt. The reaction was stirred at rt for 1 h then concentrated under reduced pressure. The resulting residue was resuspended in ethyl acetate (20 mL) and washed with 1 M HCl (3 x 20 mL), 1 M NaOH (3 x 20 mL) and brine (2 x 10 mL). The organic phase

was dried with anhydrous sodium sulfate and concentrated under reduced pressure to give a residue which was purified by column chromatography (20 – 50% ethyl acetate in *n*-hexane) to give the pure product as a white solid (294 mg, 38%). ^{1}H-NMR (700 MHz, $CDCl_3$) δ 7.34 – 7.30 (2H, m, 4-H), 7.30 – 7.26 (1H, m, 5-H or 8'-H), 7.25 – 7.21 (2H, m, 7'-H), 7.21 – 7.18 (1H, m, 5-H or 8'-H), 7.18 – 7.15 (2H, m, 3-H), 7.04 – 7.01 (2H, m, 6'-H), 5.36 – 5.30 (1H, m, 2'-H), 3.53 (2H, s, 1-H), 3.46 (2H, q, J = 6.6 Hz, 3'-H), 2.72 (2H, t, J = 6.6 Hz, 4-H). ^{13}C-NMR (176 MHz, $CDCl_3$) δ 171.0, 138.8, 134.9, 129.6, 129.2, 128.8, 128.7, 127.5, 126.6, 44.0, 40.8, 35.6; MS *m/z* [ES$^+$] 240 ([M+H]$^+$, 100%).

1-Benzyl-3,4-dihydroisoquinoline[4] 4a

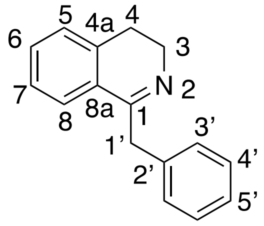

N-Phenethyl-2-phenylacetamide (281 mg, 1.17 mmol) in polyphosphoric acid (1.72 g, 17.6 mmol) was prepared and stirred at 200 °C for 6 h. The solution was diluted by the addition of water (5 mL) and the pH adjusted to 7 by addition of 12 M NaOH. The solution was sonicated to homogeneity, then extracted with diethyl ether (3 x 20 mL). The organic extracts were combined, dried with anhydrous sodium sulfate and concentrated under reduced pressure to give the product as an orange oil (229 mg, 88%). %). ^{1}H-NMR (700 MHz, $CDCl_3$) δ 7.46 (1H, d, J = 7.8 Hz, 5-H or 8-H), 7.31 – 7.25 (5H, m, Ar*H*), 7.21 – 7.15 (3H, m, Ar*H*), 4.09 (2H, s, 1'-H), 3.76 (2H, t, J = 7.6 Hz, 3-H), 2.72 (2H, t, J = 7.6 Hz, 4-H); ^{13}C-NMR (176 MHz, $CDCl_3$) δ 138.2, 138.0, 130.7, 128.9, 128.8, 128.7, 127.7, 127.0, 126.5, 125.9, 47.2, 43.0, 26.3; MS *m/z* [ES$^+$] 222 ([M+H]$^+$, 100%)

***N*-(3,4-Dimethoxyphenethyl)benzamide[3]**

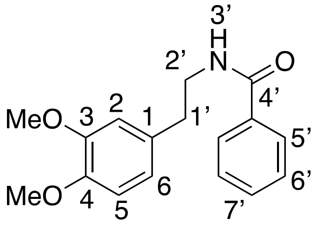

Under anhydrous condition, a solution of 3,4-dimethoxyphenethylamine (500 mg, 2.76 mmol) and triethylamine (574 μL, 4.14 mmol) in dichloromethane (10 mL) was stirred and cooled to 0 °C. Benzoyl chloride (321 μL, 2.76 mmol) was added dropwise, the solution warmed to rt and stirred for 1 h. Solvents were removed under reduced pressure and the resulting residue purified by column chromatography (20 – 50% ethyl acetate in *n*-hexane) to give the product as a white solid (789 mg, 99%). ^{1}H-NMR (700 MHz, $CDCl_3$) δ 7.70 – 7.67 (2H, m, 5'-H), 7.50 – 7.46 (1H, m, 7'-H), 7.43 – 7.39 (2H, m, 6'-H), 6.83 (1H, d, *J* = 8.1 Hz, 5-H), 6.78 (1H, dd, *J* = 8.1, 1.9 Hz, 6-H), 6.77 (1H, d, *J* = 1.9 Hz, 2-H), 6.13 (1H, m, 3'-H), 3.87 (3H, s, OCH_3), 3.84 (3H, s, OCH_3), 3.70 (2H, q, *J* = 6.6 Hz, 2'-H), 2.88 (2H, t, *J* = 6.6 Hz, 1'-H); ^{13}C-NMR (176 MHz, $CDCl_3$) δ 167.6, 149.2, 147.9, 134.8, 131.6, 131.5, 128.7, 126.9, 120.8, 112.1, 111.6, 56.1, 56.0, 41.4, 35.4; MS *m/z* [ES$^+$] 286 ([M+H]$^+$, 100%)

6,7-Dimethoxy-1-phenyl-3,4-dihydroisoquinoline[3] 5a

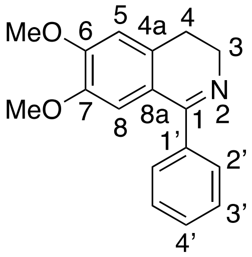

Under anhydrous conditions, a solution of *N*-(3,4-dimethoxyphenethyl)benzamide (680 mg, 2.28 mmol) in ethyl acetate (10 mL) was prepared and stirred. $POCl_3$ (423 μL, 4.56 mmol) was added dropwise over 5 min and the reaction heated under reflux for 6 h. The reaction was then quenched by the dropwise addition of water (5 mL). The product was extracted into 2 M HCl (3 x 20 mL). The pH of the combined aqueous extractions was then adjusted to 13 by addition of 6 M NaOH, and extracted into dichloromethane (3 x 20 mL). The dichloromethane layers were then combined, dried with anhydrous sodium sulfate and concentrated under reduced pressure to give the product as a brown oil (571 mg, 94%). %). ^{1}H-NMR (700 MHz, $CDCl_3$) δ 7.62 – 7.58 (2H, m, 2'-H), 7.46 – 7.39 (3H, m, 3'-H and 4'-H), 7.80 – 7.26 (2H, m, 5-H and 8-H), 3.94 (3H, s, OCH_3), 3.84 – 3.79 (2H, t, *J* = 7.6 Hz, 3-H), 3.72 (3H, s, OCH_3), 2.73 (2H, t, *J* = 7.6 Hz, 4-H); ^{13}C-NMR (150 MHz, $CDCl_3$) δ 167.0, 151.1, 147.2, 132.8, 129.6, 128.9, 128.3, 121.6, 111.7, 110.3, 56.3, 56.2, 47.7, 26.1; MS *m/z* [ES$^+$] 268 ([M+H]$^+$, 100%).

6,7-Dimethoxy-1-phenyl-1,2,3,4-tetrahydroisoquinoline[3] 5b

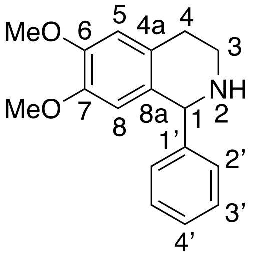

Under anhydrous conditions, a solution of 6,7-dimethoxy-1-phenyl-3,4-dihydroisoquinoline (117 mg, 0.44 mmol) in methanol (10 mL) was prepared and stirred. Sodium borohydride (33 mg, 0.88 mmol) was added, and the reaction stirred at rt for 2 h. The reaction was quenched by the dropwise addition of water (2 mL) then the pH of the reaction adjusted to 10 by addition of 6 M NaOH. The product was extracted into ethyl acetate (3 x 20 mL), then the organic layers combined, dried with anhydrous sodium sulfate and concentrated under reduced pressure to give the product as a white solid (114 mg, 95%). ^{1}H-NMR (700 MHz, $CDCl_3$) δ 7.36 – 7.32 (2H, m, 2'-H), 7.31 – 7.27 (3H, m, 3'-H and 4'-H), 6.63 (1H, s, 8-H), 6.23 (1H, s, 5-H), 5.13 (1H, s, 1-H), 3.87 (3H, s, OCH_3), 3.64 (3H, s, OCH_3), 3.23 – 3.18 (1H, m, 3-*H*H), 3.05 – 2.96 (2H, m, 3-H*H* and 4-*H*H), 3.84 – 2.79 (1H, m, 4-H*H*); ^{13}C-NMR (176 MHz, $CDCl_3$) δ 148.1, 147.5, 129.4, 128.9, 128.7, 128.3, 128.0, 127.1, 111.5, 111.0, 56.0, 56.0, 53.6, 41.2, 28.4; MS *m/z* [ES$^+$] 270 ([M+H]$^+$, 100%).

NMR spectra

N-Phenethylbenzamide

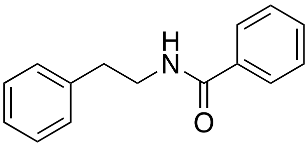

^{1}H-NMR (700 MHz)

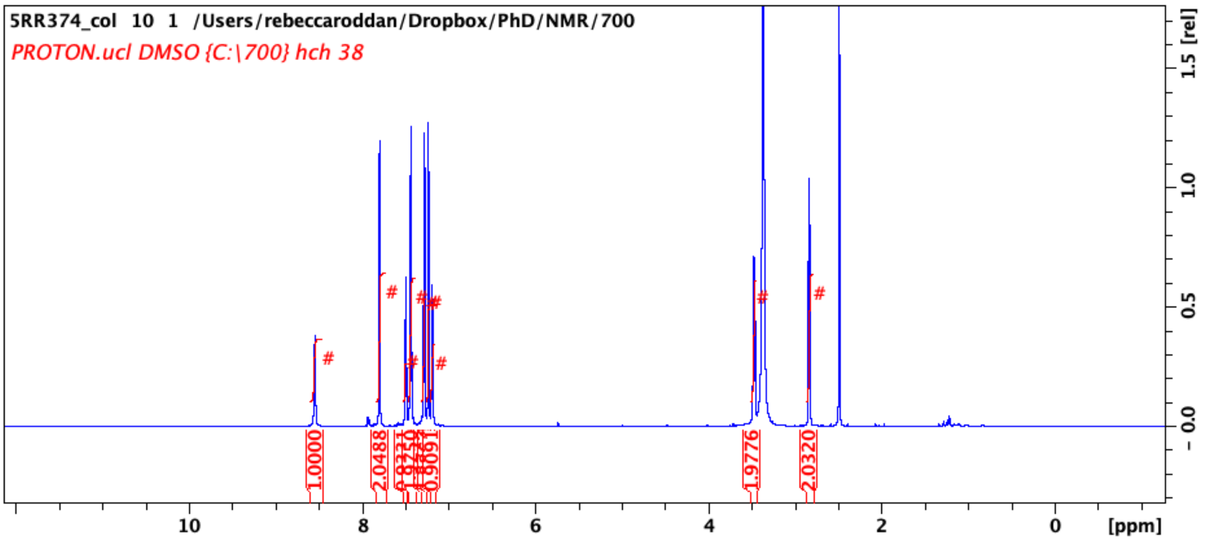

^{1}H-NMR (700 MHz): 8.7 – 7.0 ppm

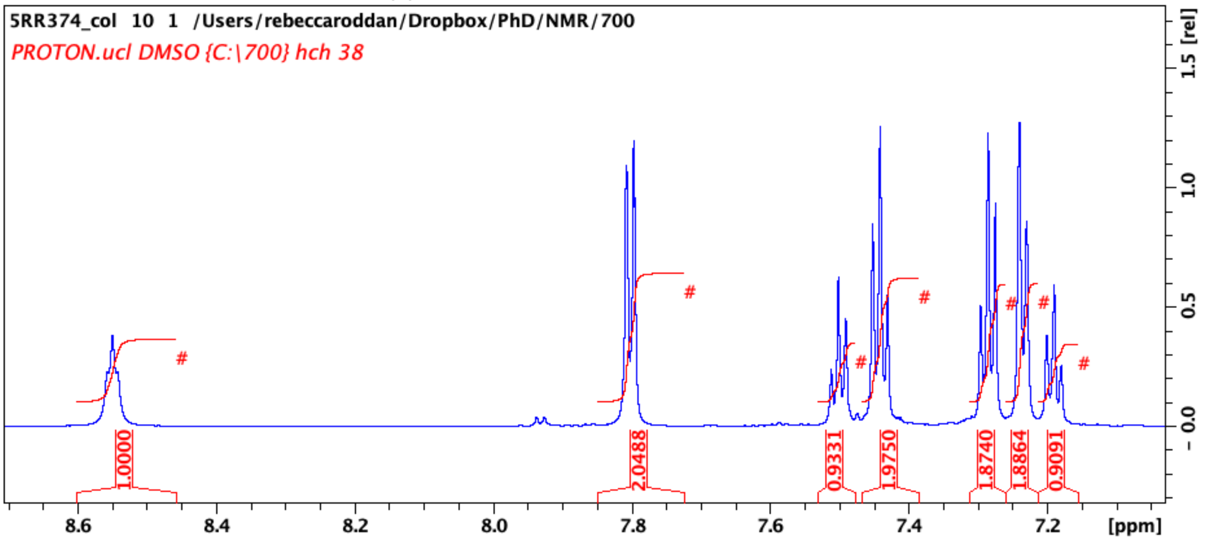

^{1}H-NMR (700 MHz): 3.9 – 2.2 ppm

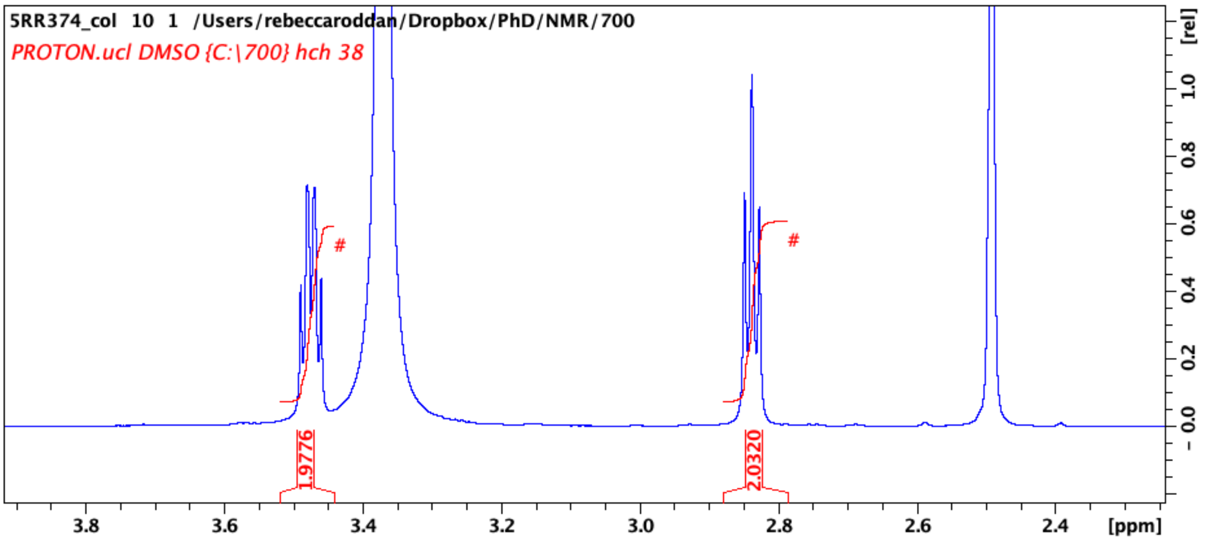

^{13}C-NMR (176 MHz)

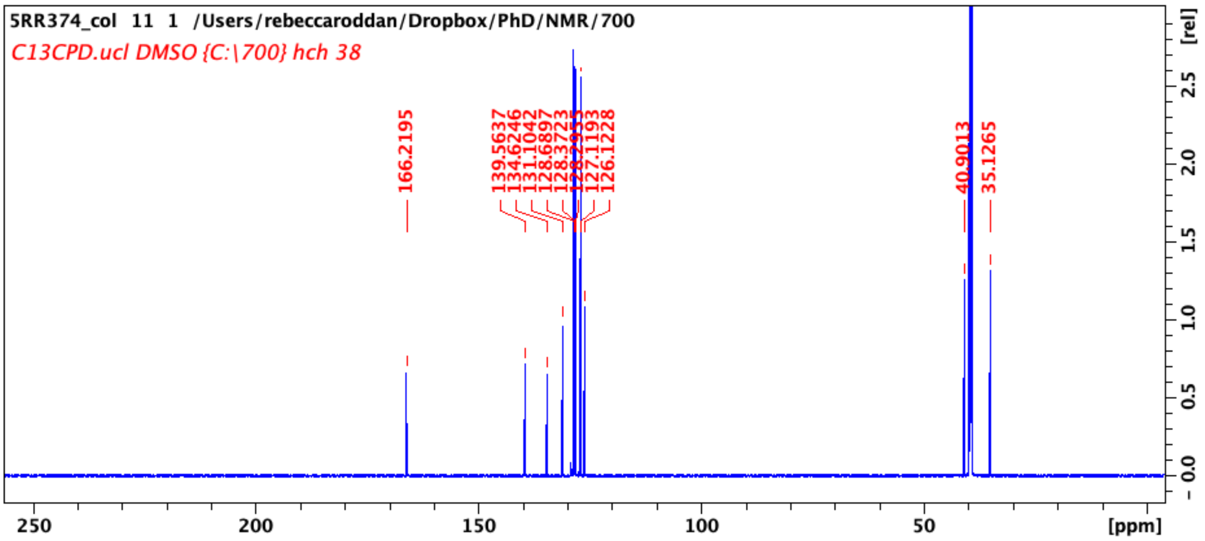

1-Phenyl-3,4-dihydroisoquinoline 1a

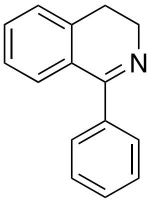

^{1}H-NMR (700 MHz)

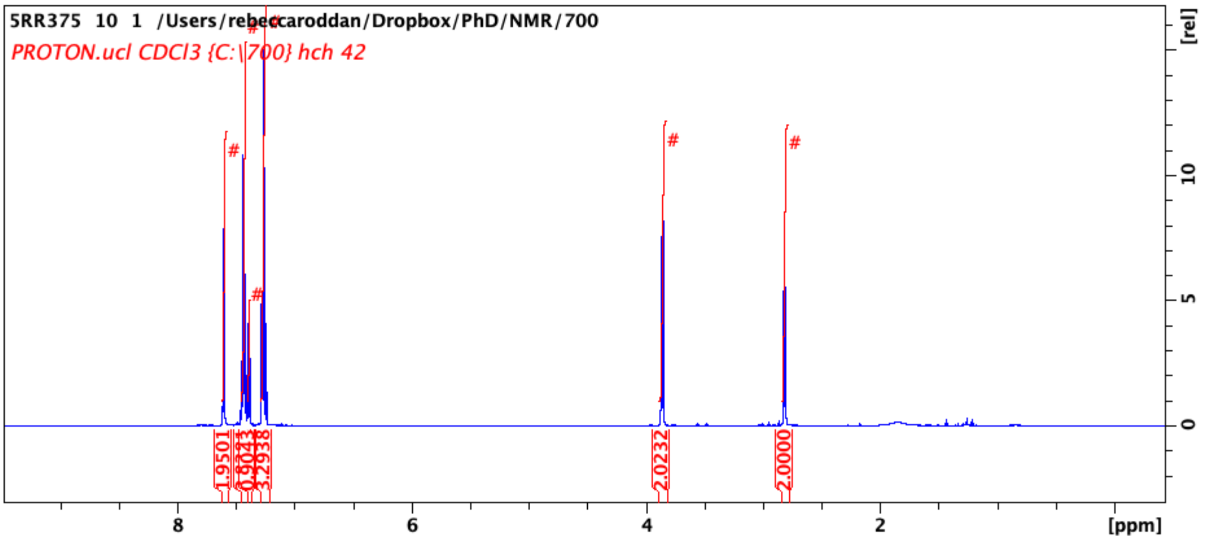

^{1}H-NMR (700 MHz): 7.7 – 7.2 ppm

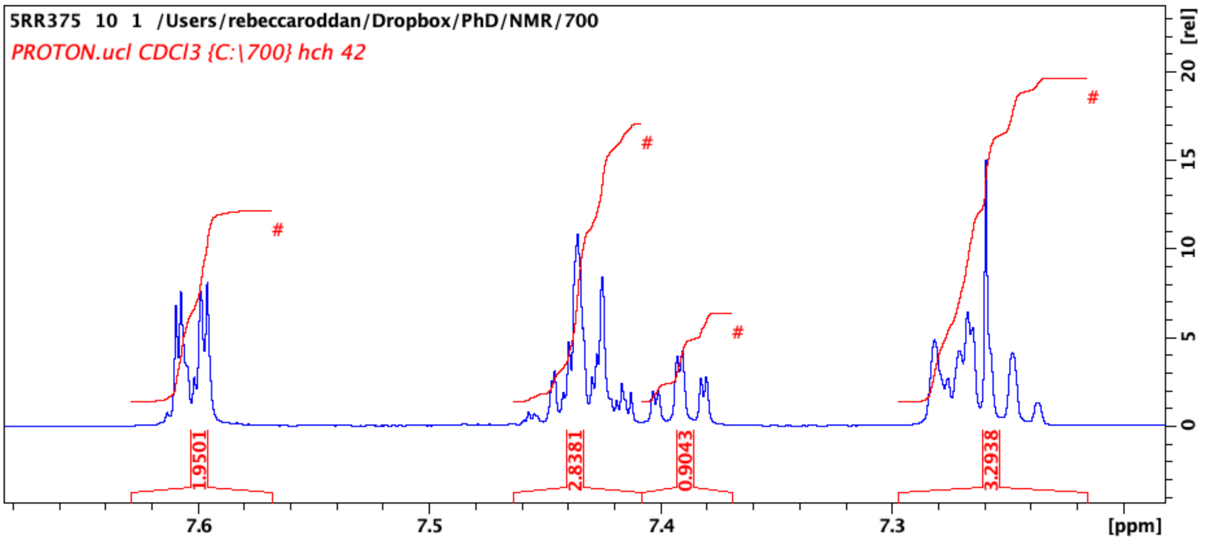

^{1}H-NMR (700 MHz): 3.9 – 2.8 ppm

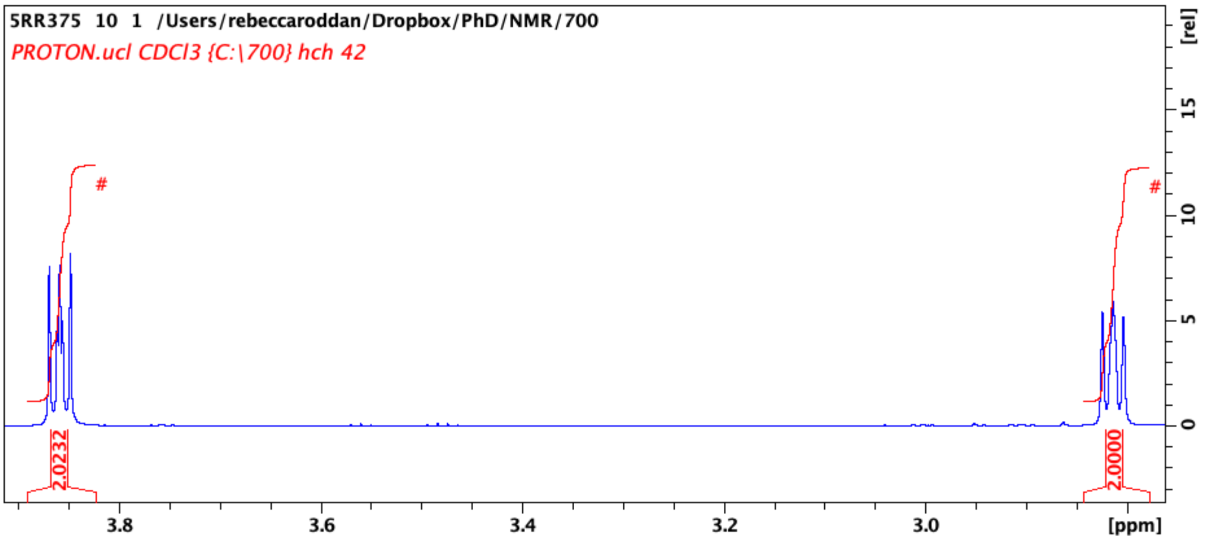

^{13}C-NMR (176 MHz)

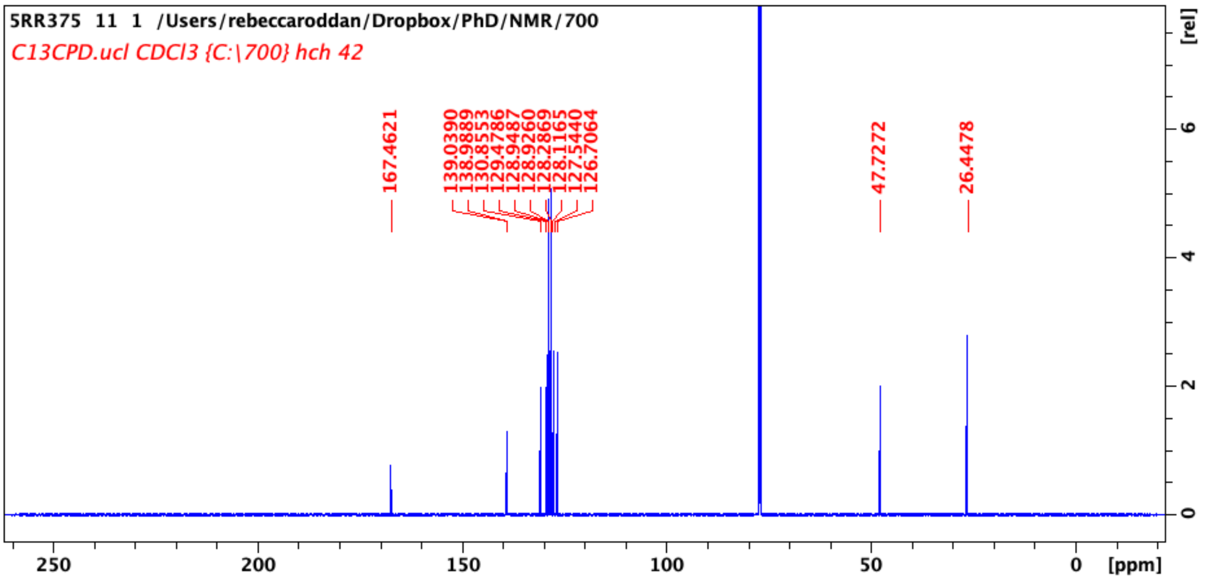

N-(3,4-Dimethoxyphenethyl)benzo[*d*][1,3]dioxole-5-carboxamide

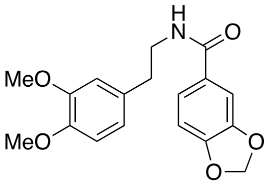

^{1}H-NMR (700 MHz)

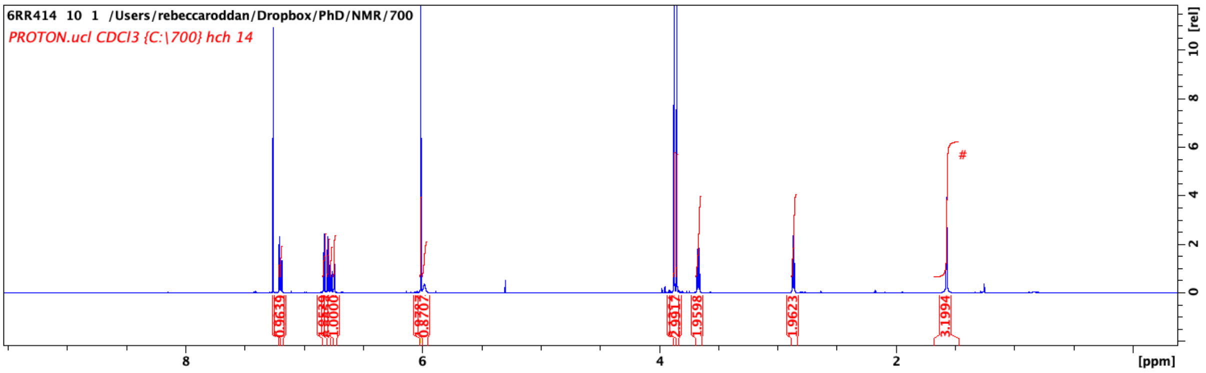

^{1}H-NMR (700 MHz): 7.3 – 5.9 ppm

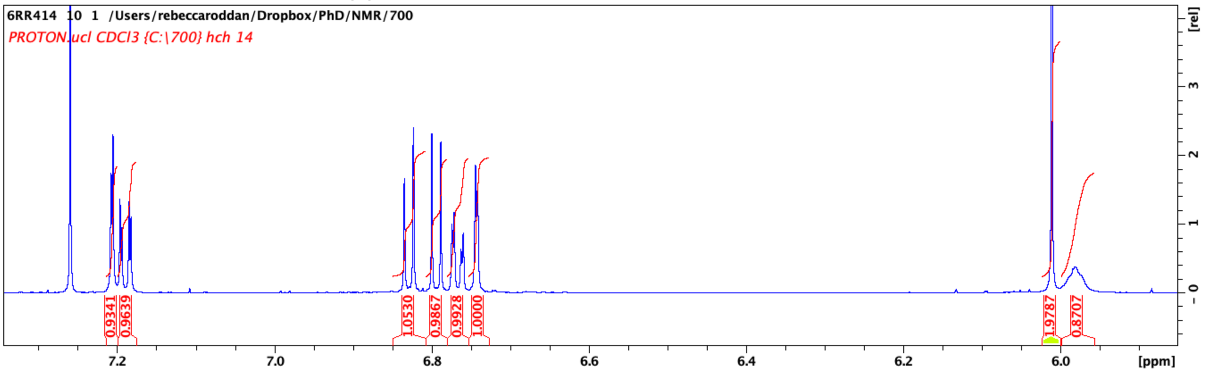

^{1}H-NMR (700 MHz): 4.1 – 2.6 ppm

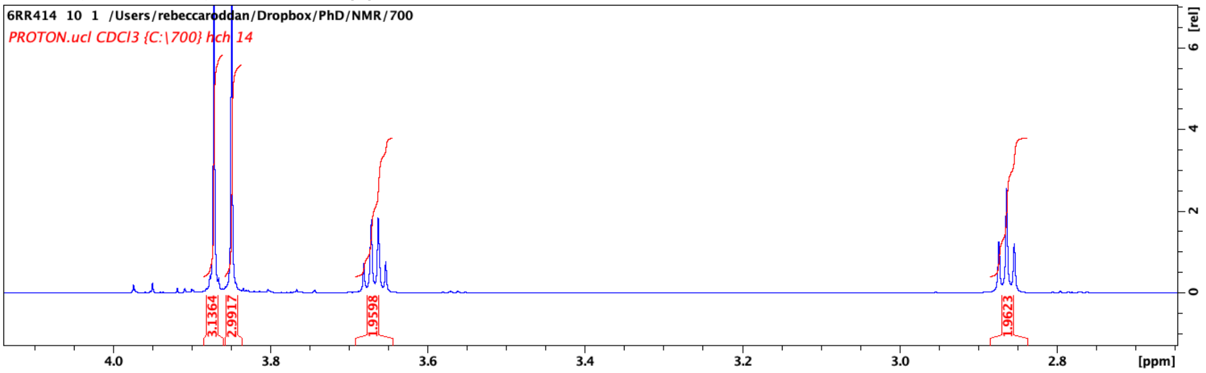

^{13}C-NMR (176 MHz)

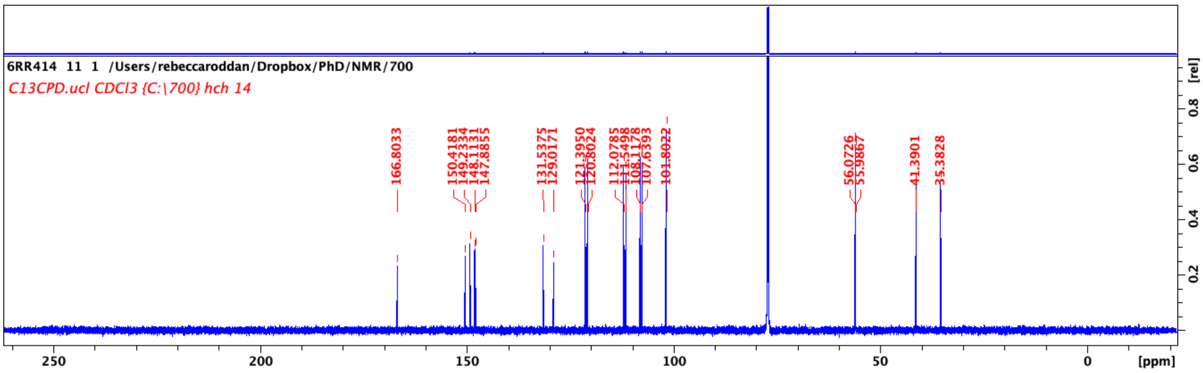

1-(Benzo[*d*][1,3]dioxol-5-yl)-6,7-dimethoxy-3,4-dihydroisoquinoline 2a

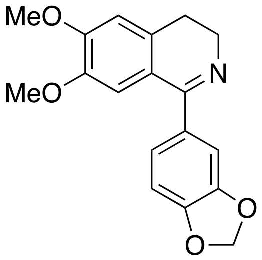

^{1}H-NMR (700 MHz)

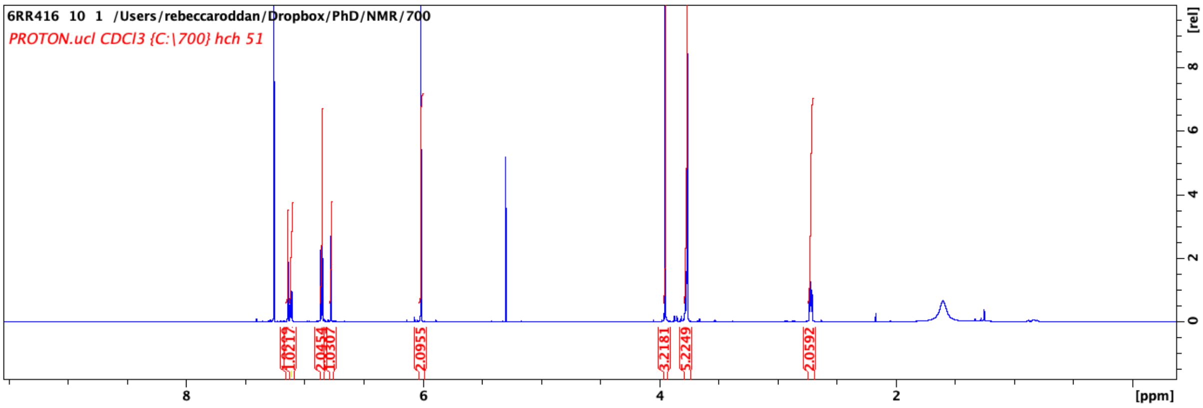

^{1}H-NMR (700 MHz): 7.3 – 5.9 ppm

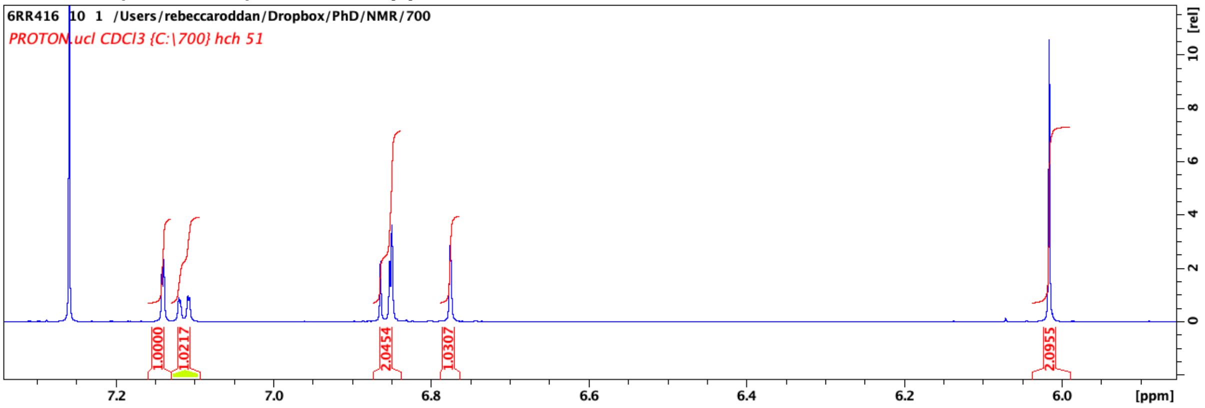

^{1}H-NMR (700 MHz): 4.0 – 2.6 ppm

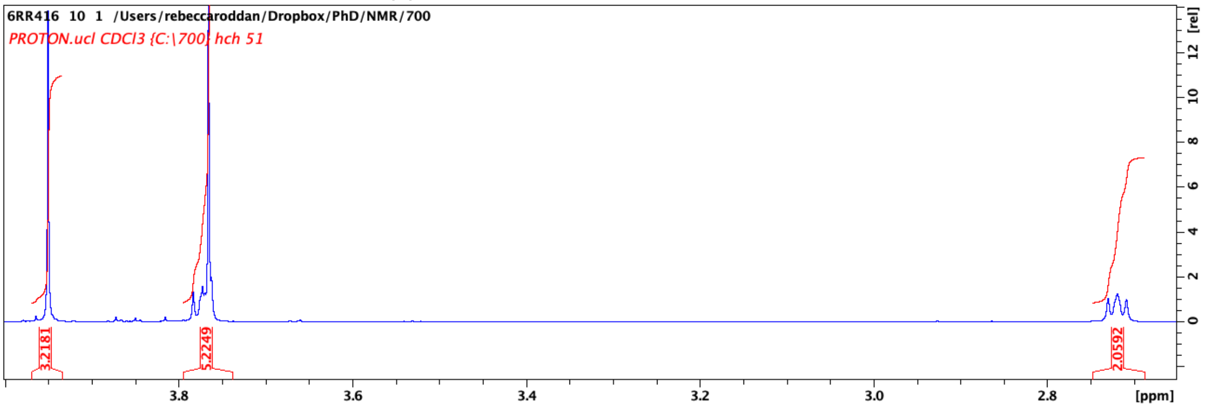

^{13}C-NMR (176 MHz)

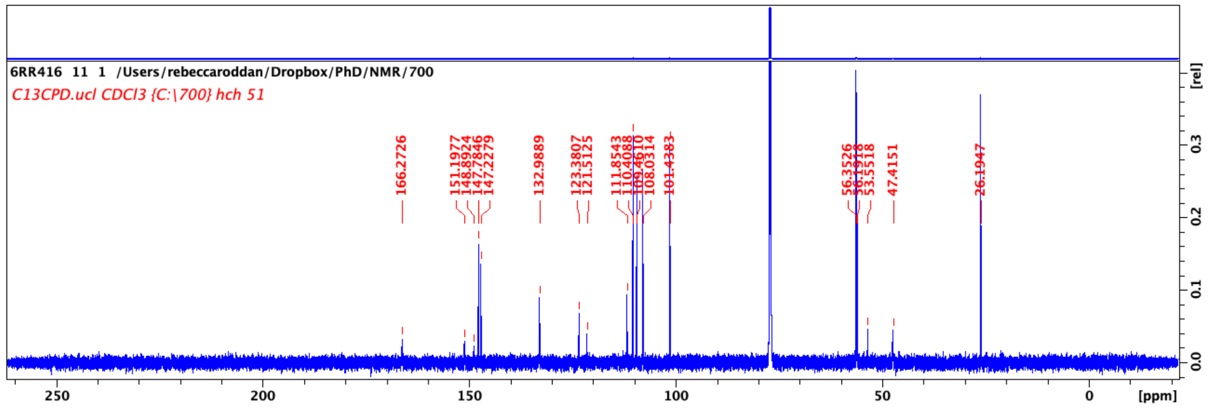

1-(Benzo[*d*][1,3]dioxol-5-yl)-6,7-dimethoxy-1,2,3,4-tetrahydroisoquinoline 2b

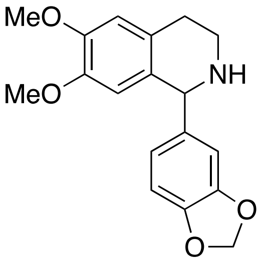

^{1}H-NMR (700 MHz)

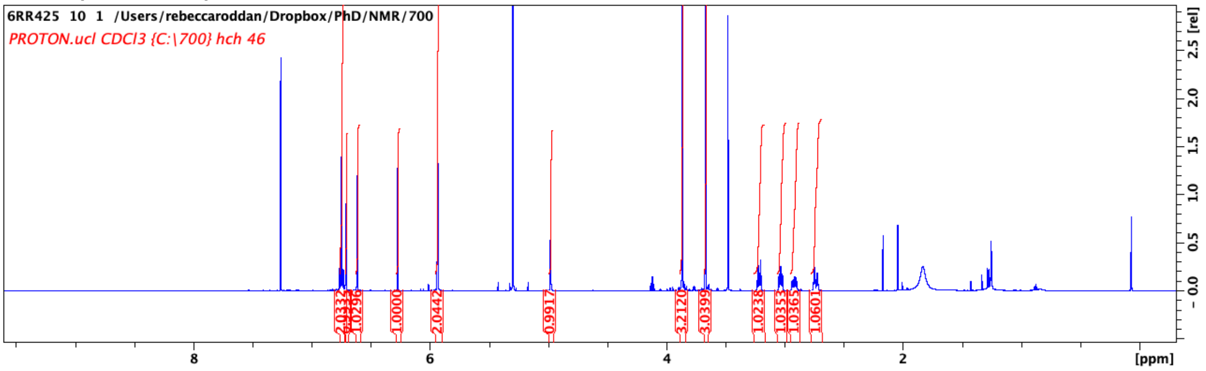

^{1}H-NMR (700 MHz): 7.1 – 4.6 ppm

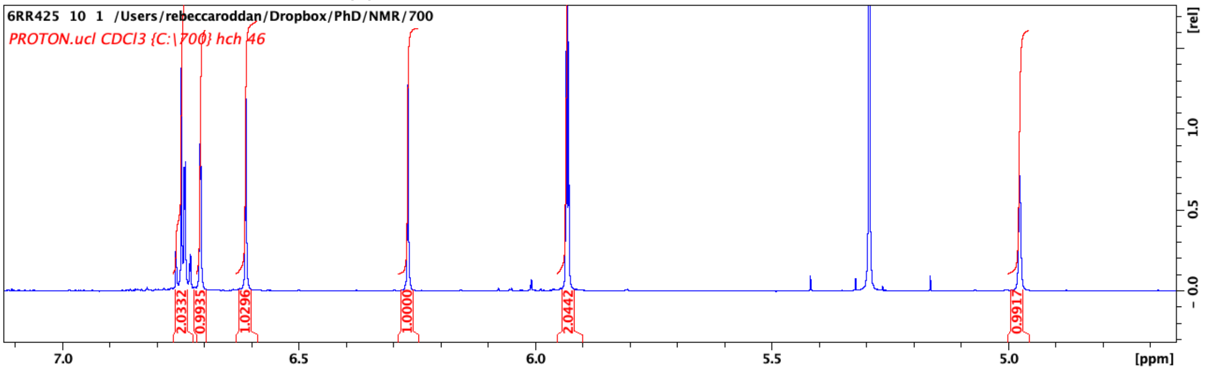

^{1}H-NMR (700 MHz): 3.9 – 2.7 ppm

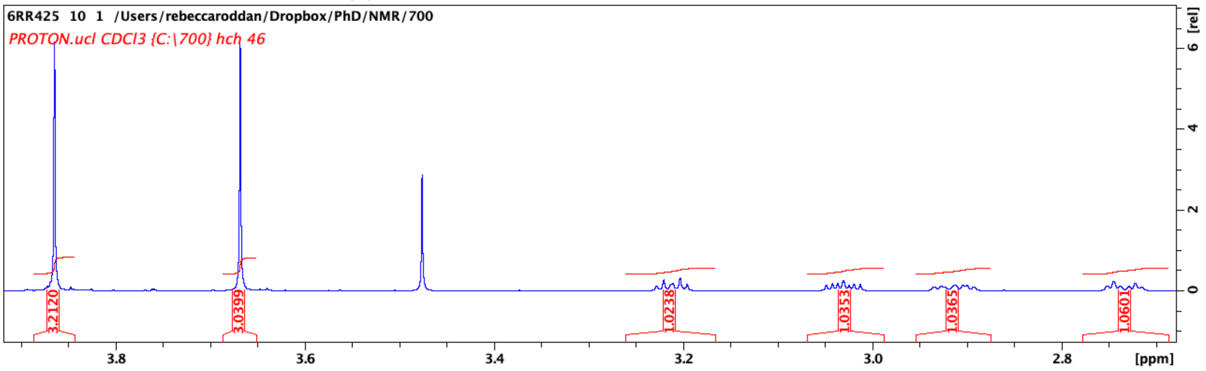

^{13}C-NMR (176 MHz)

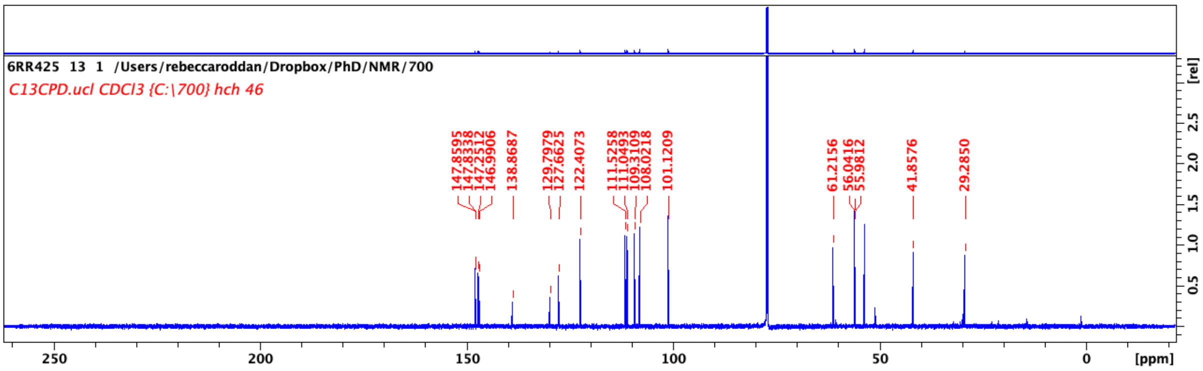

4-Fluoro-*N*-phenethylbenzamide

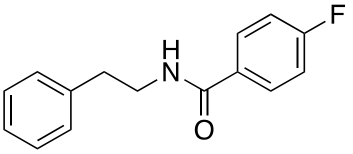

^{1}H-NMR (700 MHz)

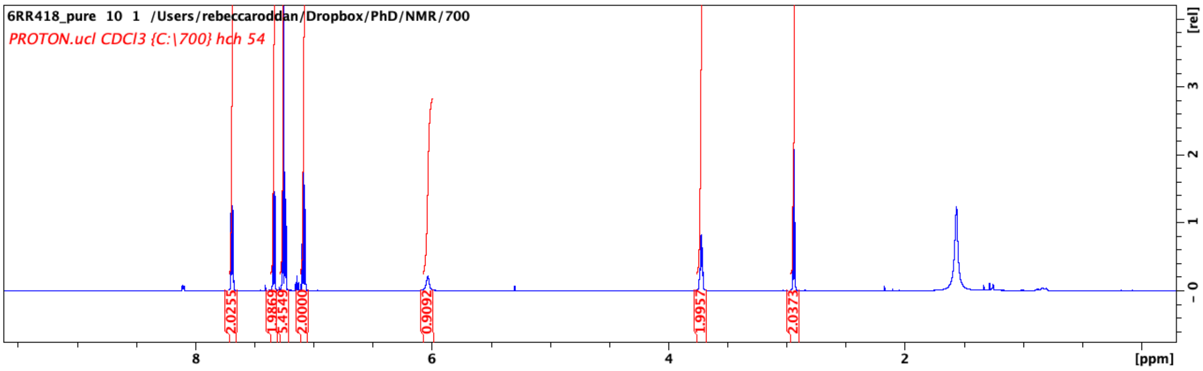

^{1}H-NMR (700 MHz): 7.8 – 6.9 ppm

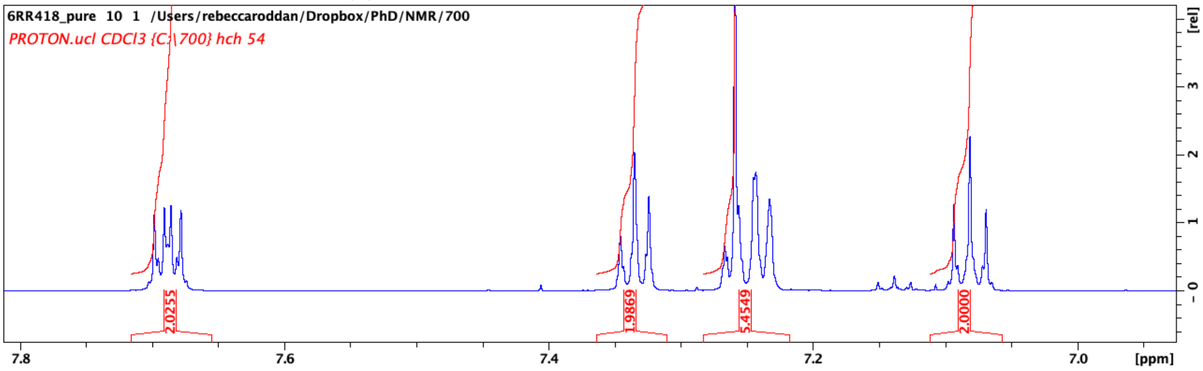

^{1}H-NMR (700 MHz): 3.8 – 2.8 ppm

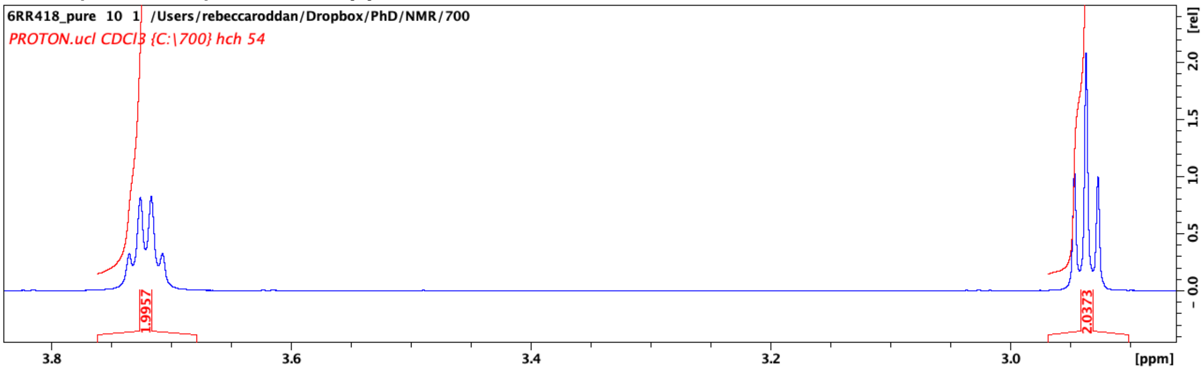

^{13}C-NMR (176 MHz)

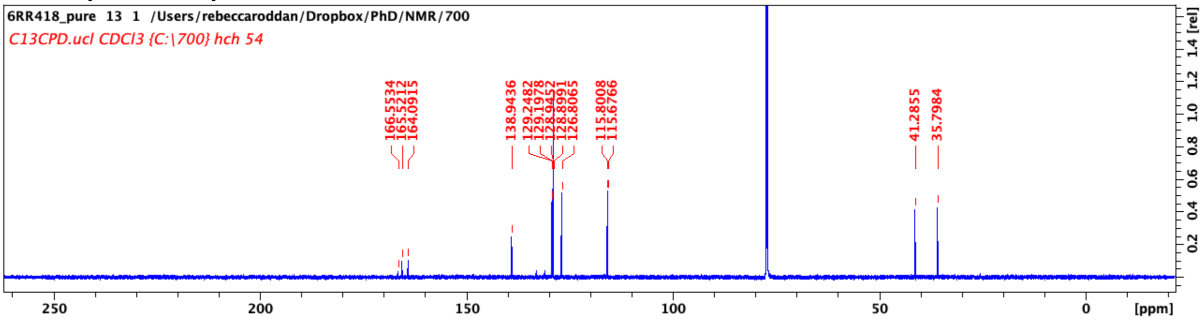

1-(4-Fluorophenyl)-3,4-dihydroisoquinoline 3a

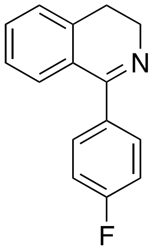

^{1}H-NMR (700 MHz)

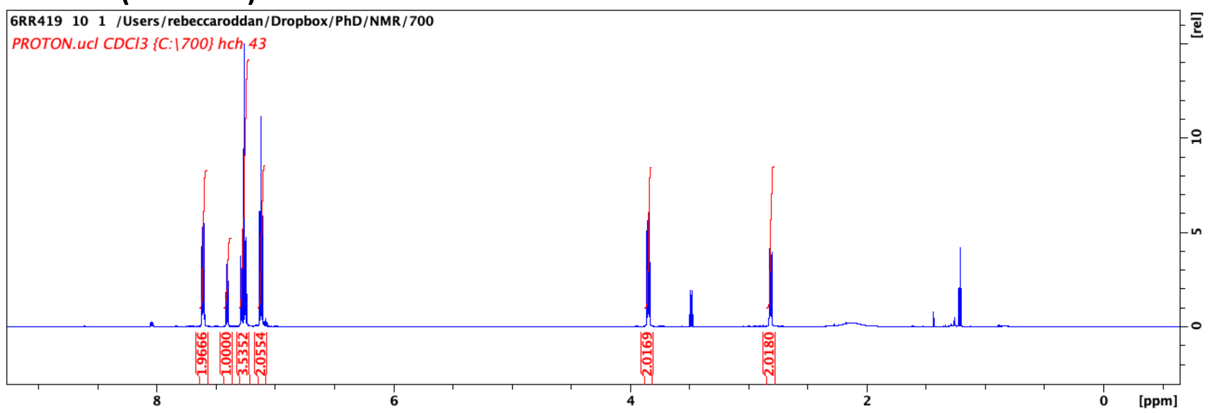

^{1}H-NMR (700 MHz): 7.7 – 7.0 ppm

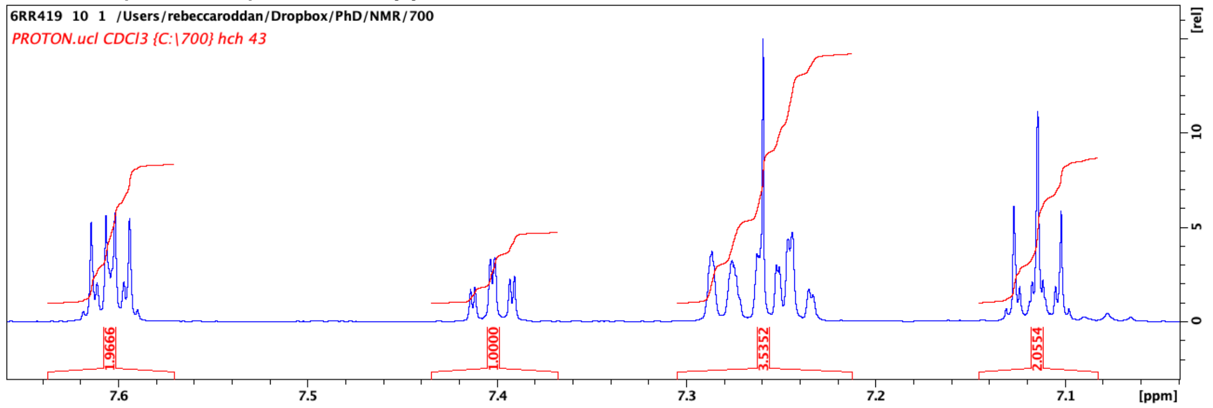

^{1}H-NMR (700 MHz): 3.9 – 2.7 ppm

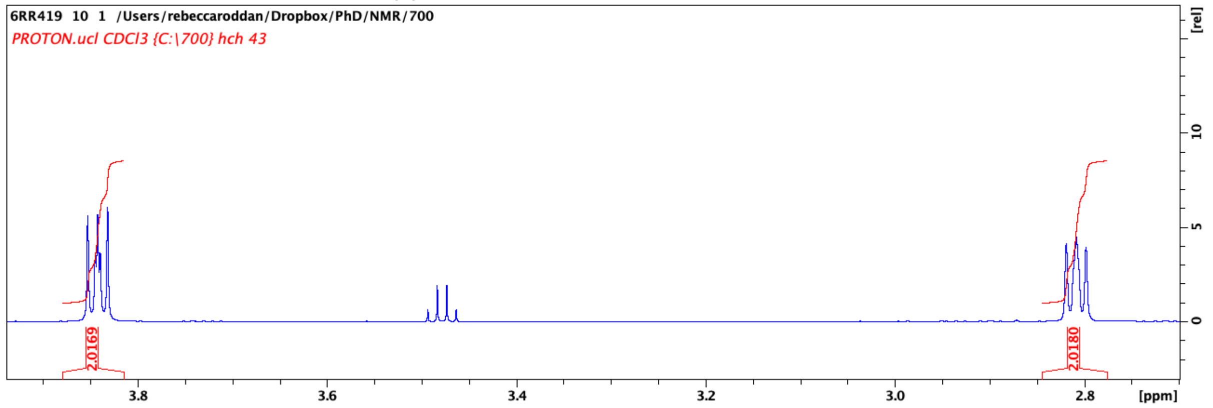

^{13}C-NMR (176 MHz)

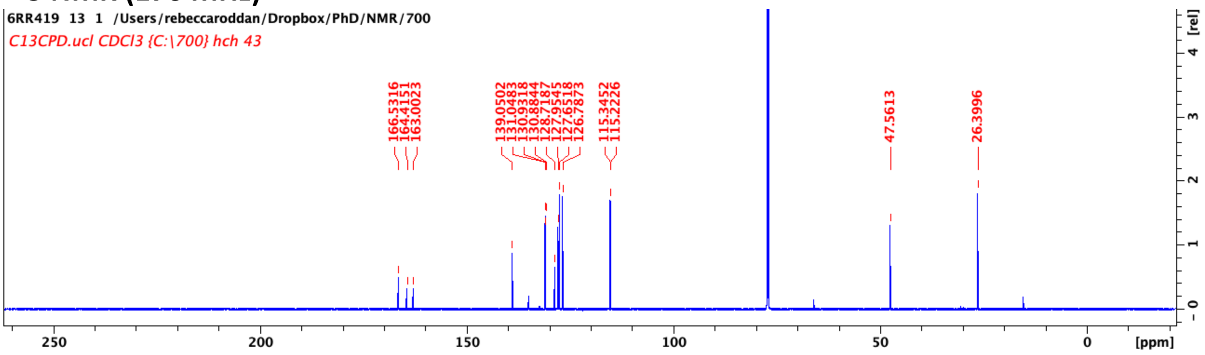

1-(4-Fluorophenyl)-1,2,3,4-tetrahydroisoquinoline 3b

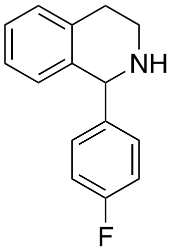

^{1}H-NMR (700 MHz)

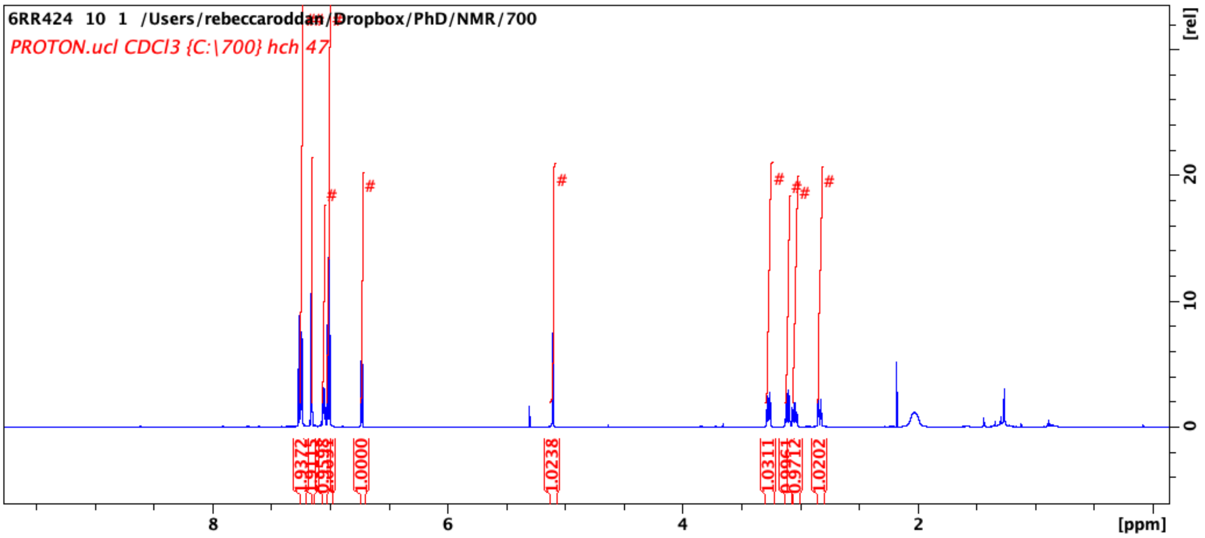

^{1}H-NMR (700 MHz): 7.3 – 6.7 ppm

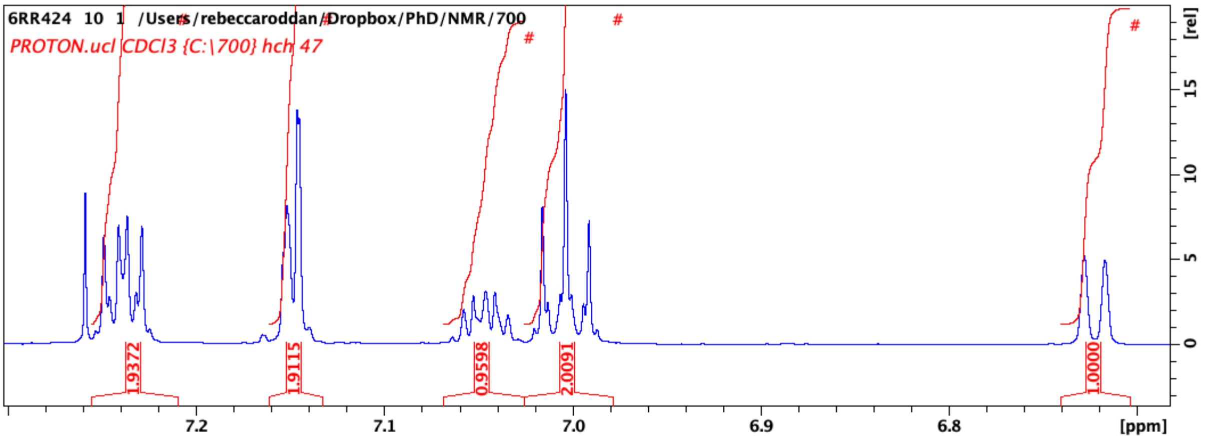

^{1}H-NMR (700 MHz): 3.4 – 2.7 ppm

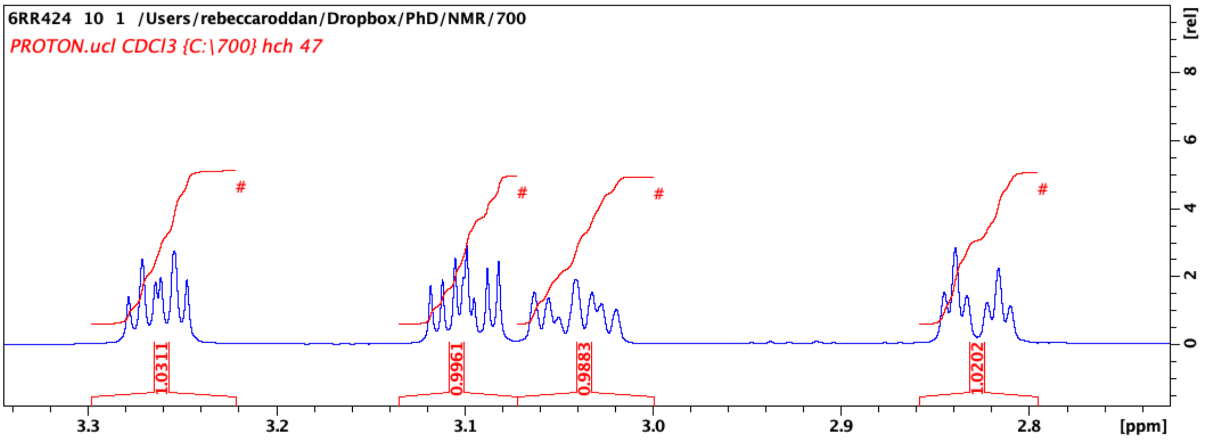

^{13}C-NMR (176 MHz)

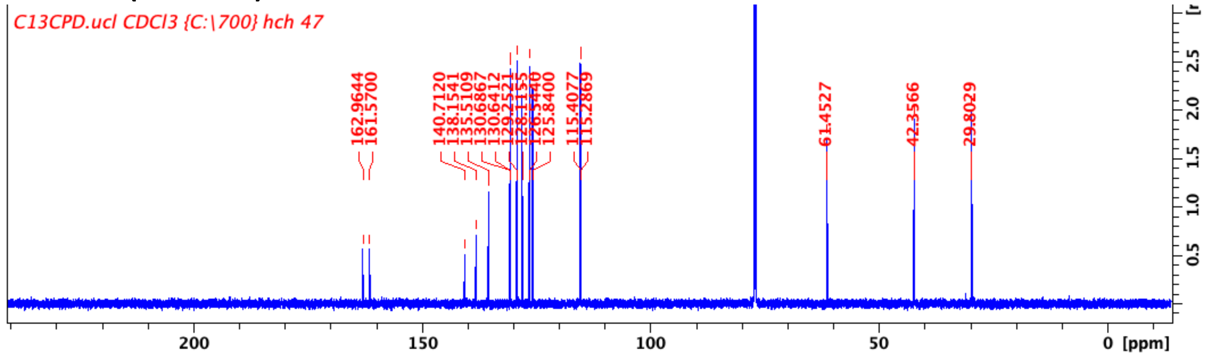

***N*-Phenethyl-2-phenylacetamide**

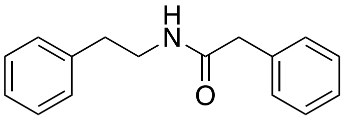

^{1}H-NMR (700 MHz)

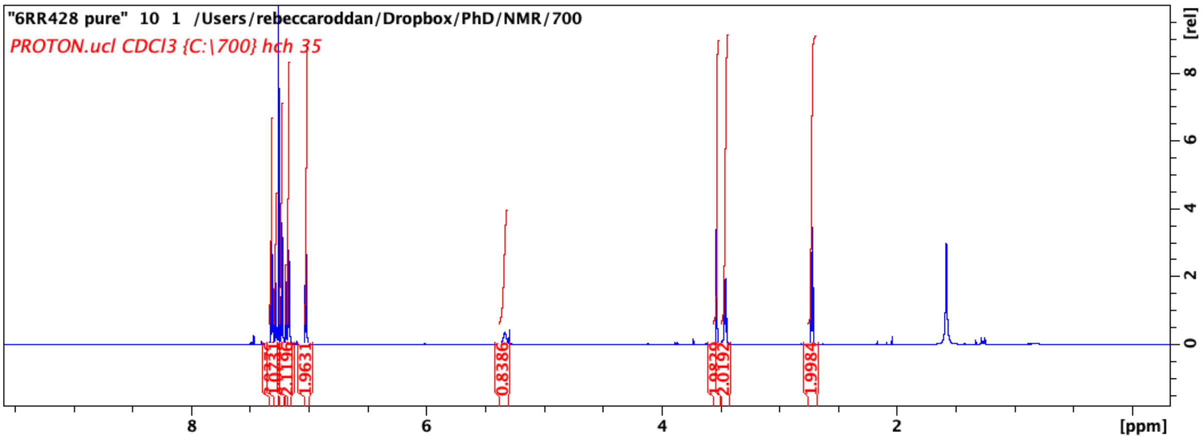

^{1}H-NMR (700 MHz): 7.6 – 6.8 ppm

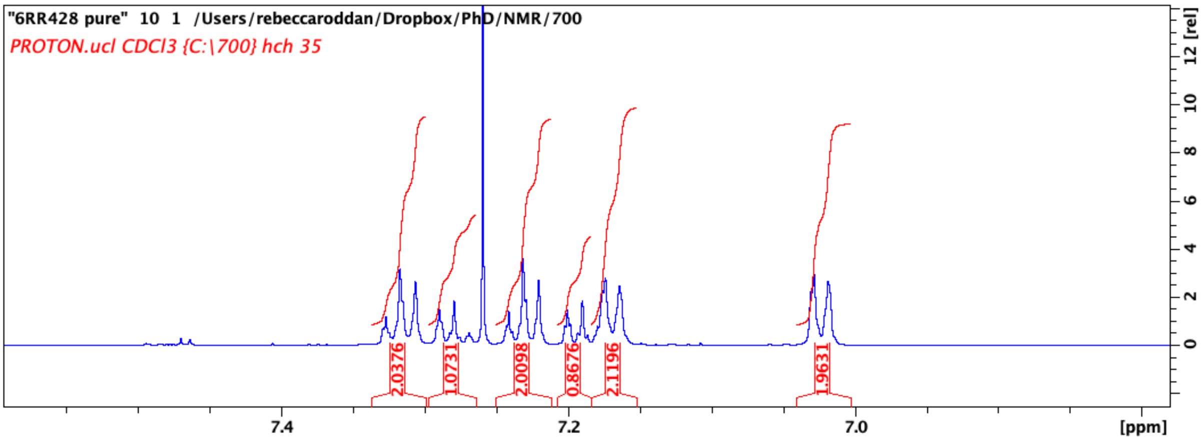

^{1}H-NMR (700 MHz): 3.6 – 2.6 ppm

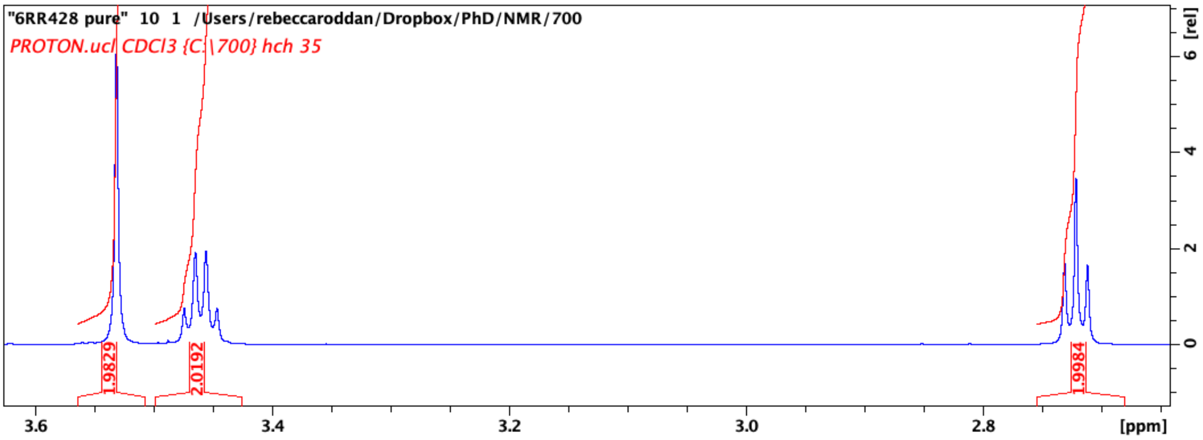

^{13}C-NMR (176 MHz)

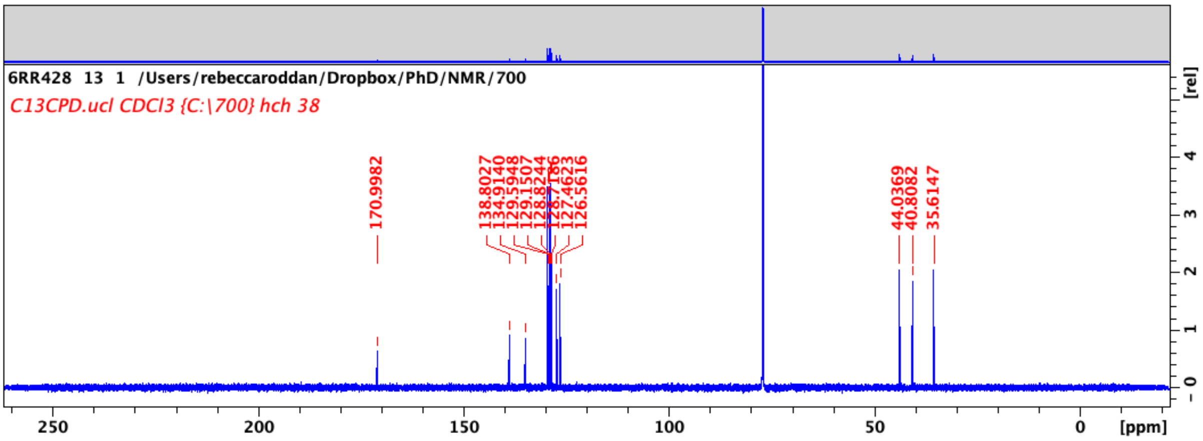

1-Benzyl-3,4-dihydroisoquinoline 4a

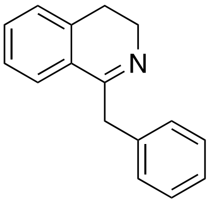

^{1}H-NMR (700 MHz)

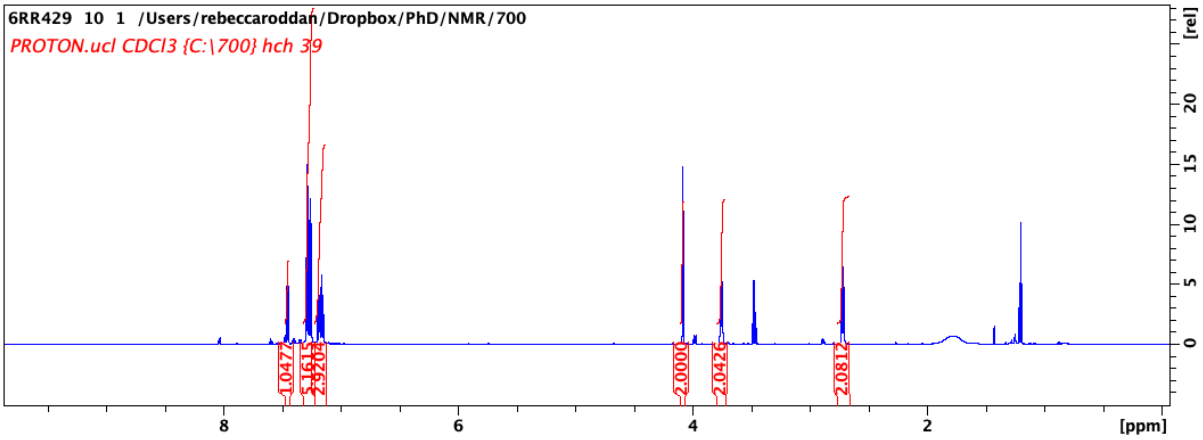

^{1}H-NMR (700 MHz): 7.5 – 7.1 ppm

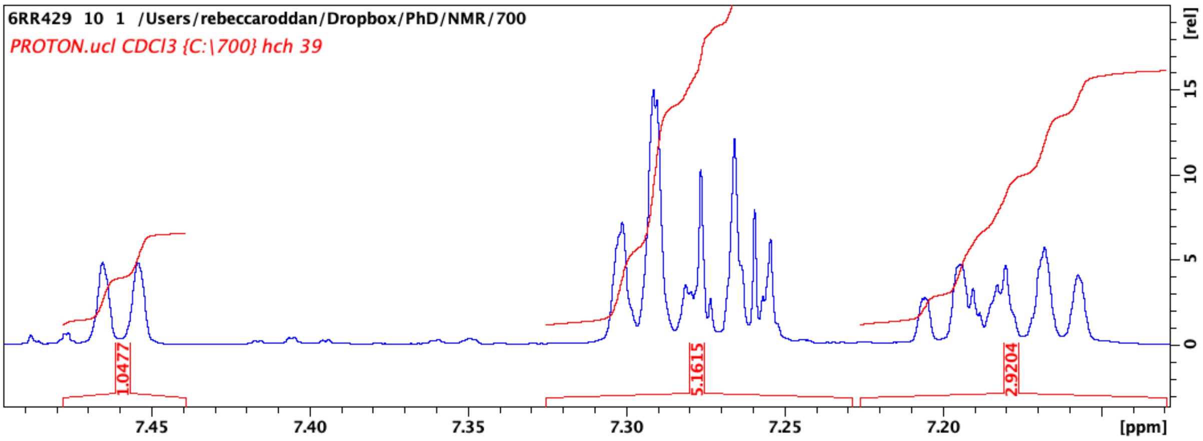

^{1}H-NMR (700 MHz): 4.2 – 2.6 ppm

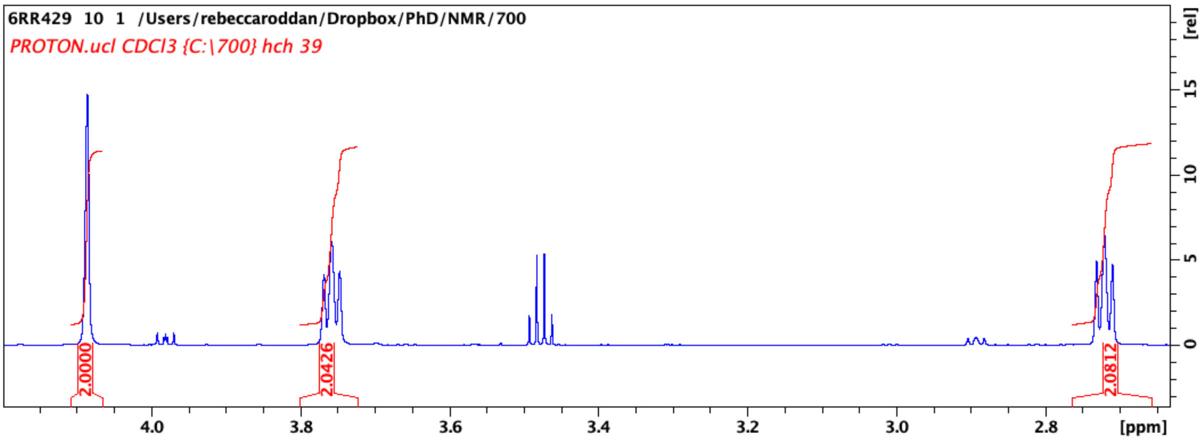

^{13}C-NMR (700 MHz)

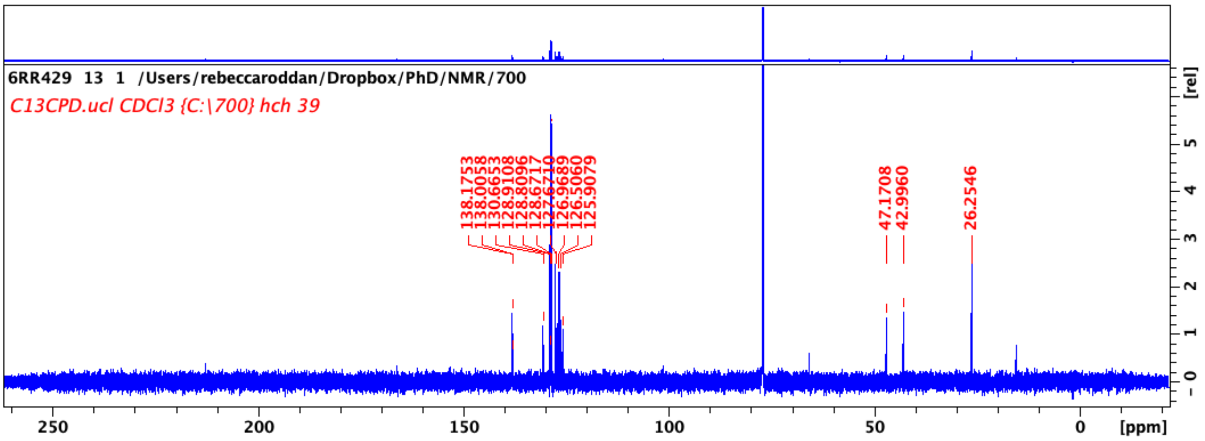

N-(3,4-Dimethoxyphenethyl)benzamide

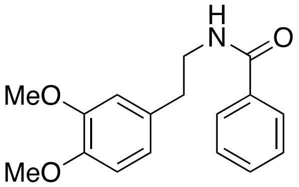

^{1}H-NMR (700 MHz)

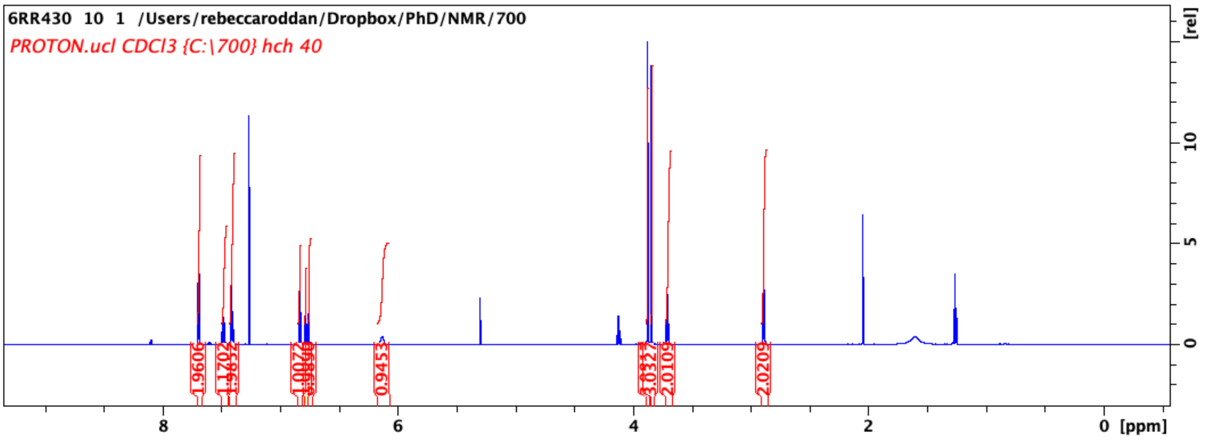

^{1}H-NMR (700 MHz): 7.8 – 6.6 ppm

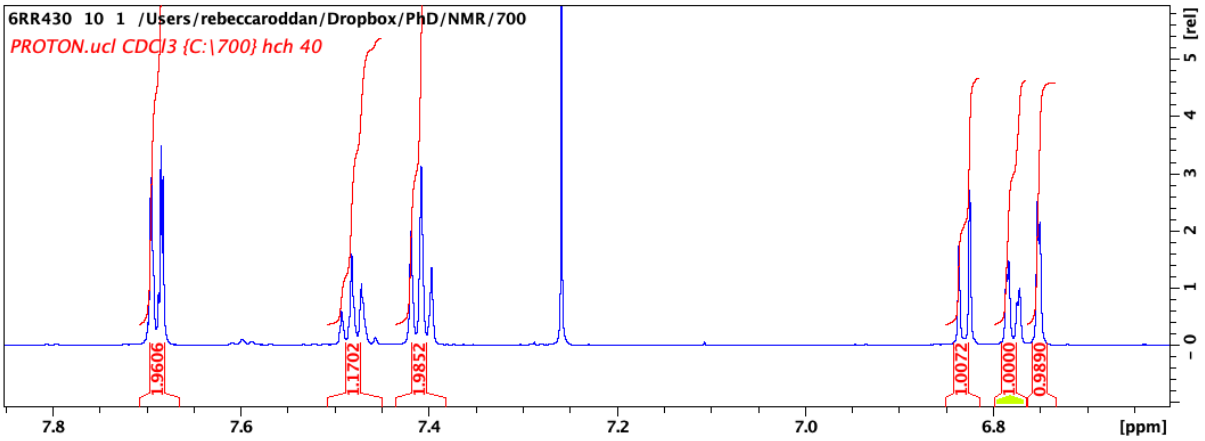

^{1}H-NMR (700 MHz): 4.0 – 2.7 ppm

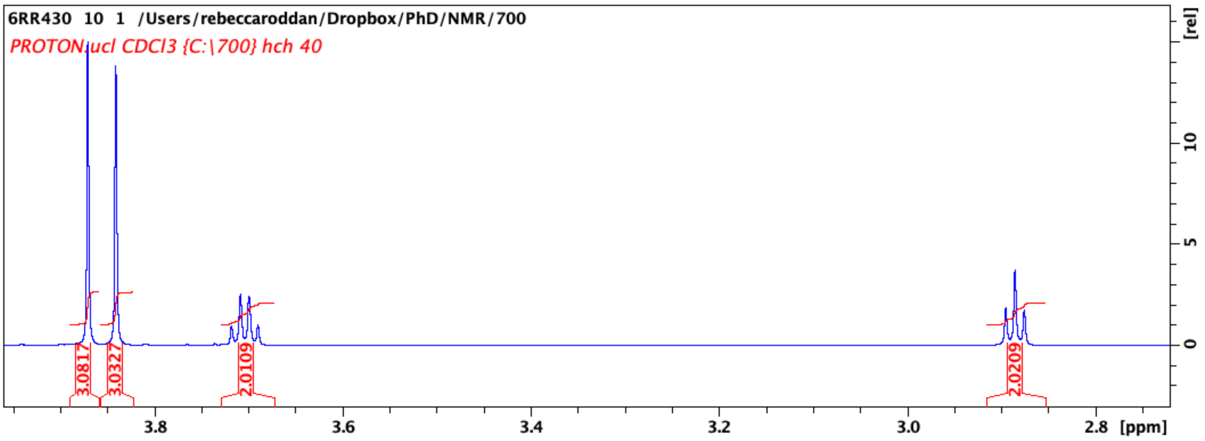

^{13}C-NMR (176 MHz)

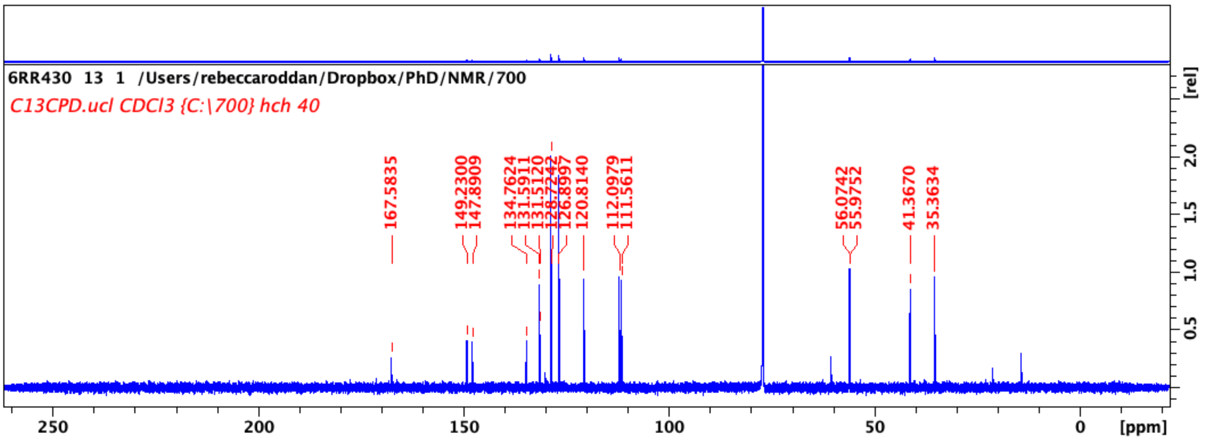

6,7-Dimethoxy-1-phenyl-3,4-dihydroisoquinoline 5a

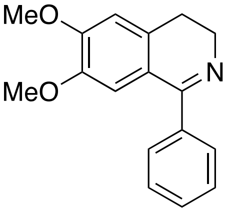

^{1}H-NMR (700 MHz)

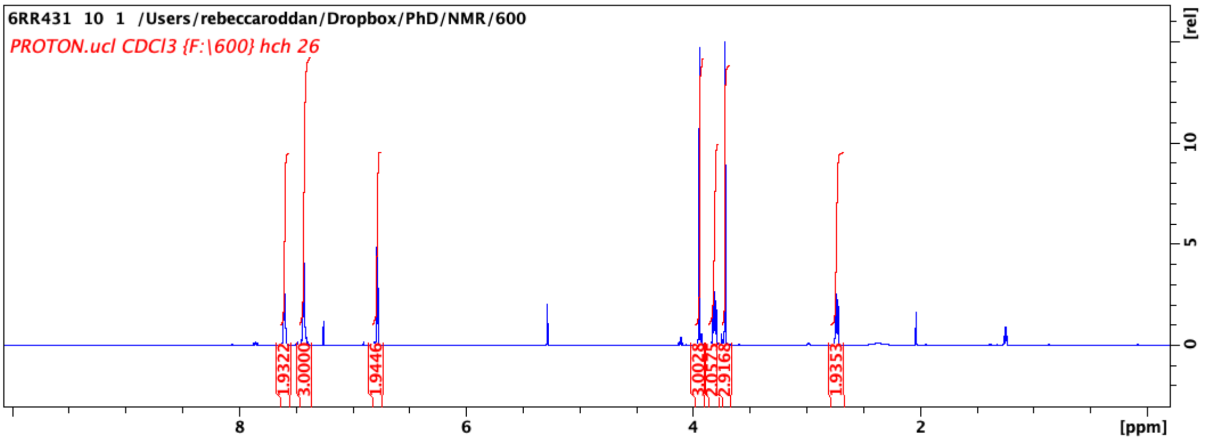

^{1}H-NMR (700 MHz): 7.8 – 6.6 ppm

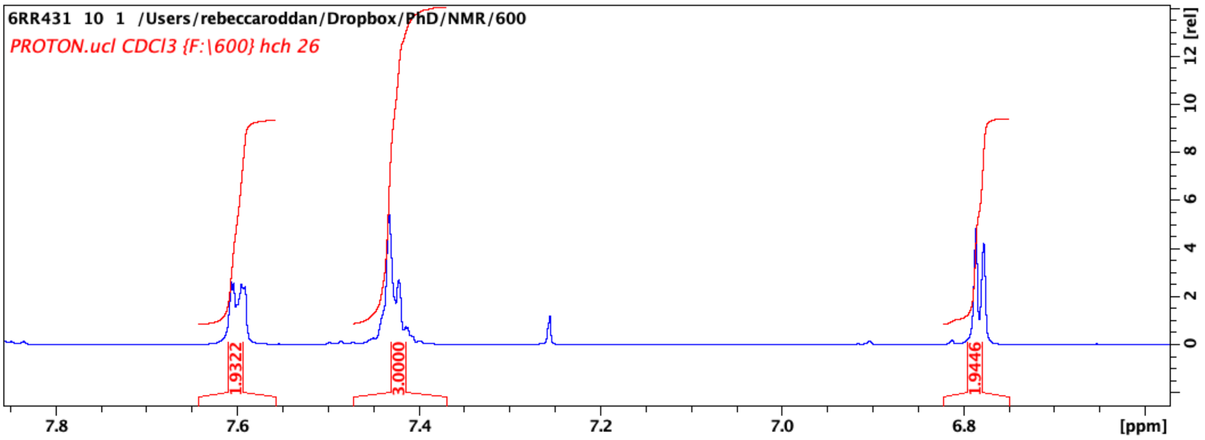

^{1}H-NMR (700 MHz): 4.0 – 2.6 ppm

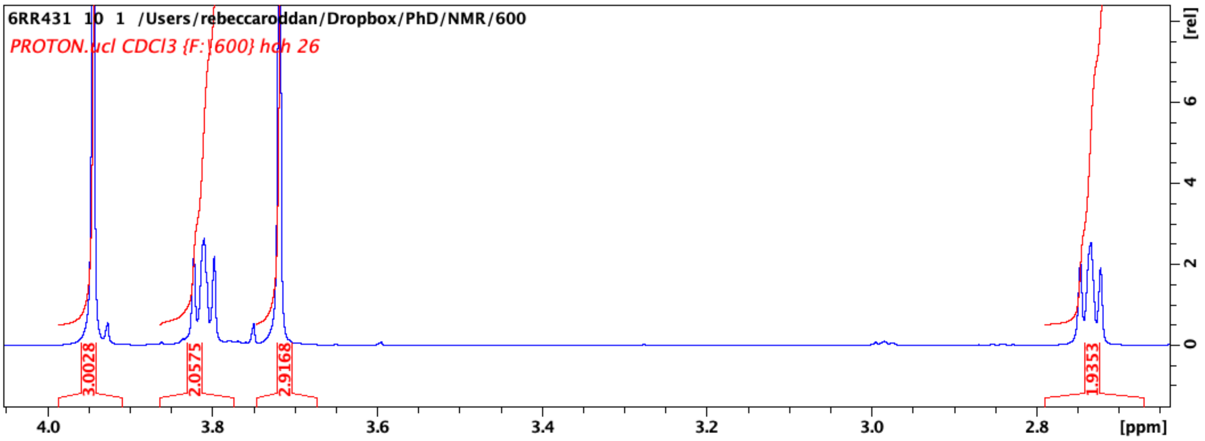

^{13}C-NMR (176 MHz)

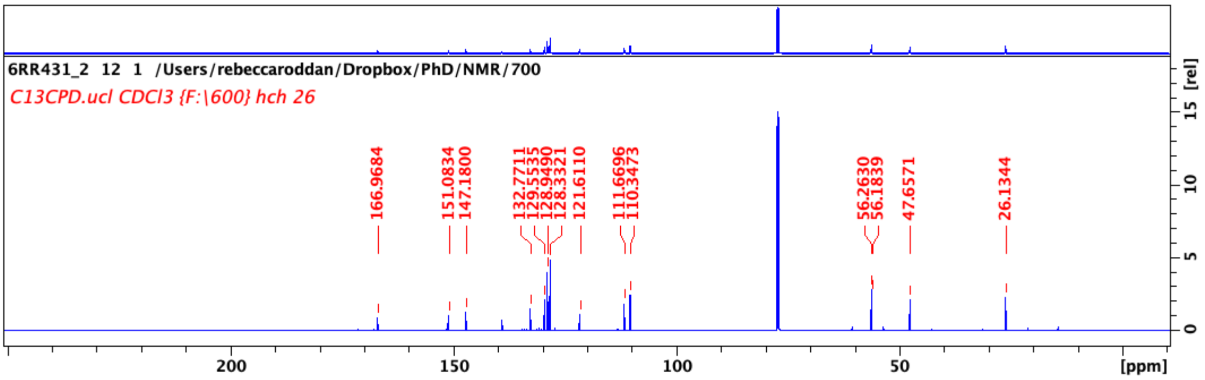

6,7-Dimethoxy-1-phenyl-1,2,3,4-tetrahydroisoquinoline 5b

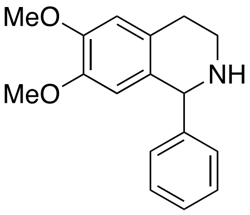

^{1}H-NMR (700 MHz)

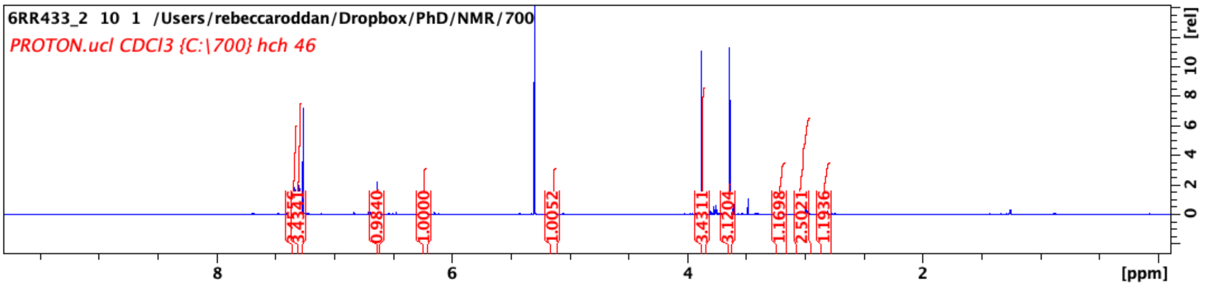

^{1}H-NMR (700 MHz): 7.6 – 6.0 ppm

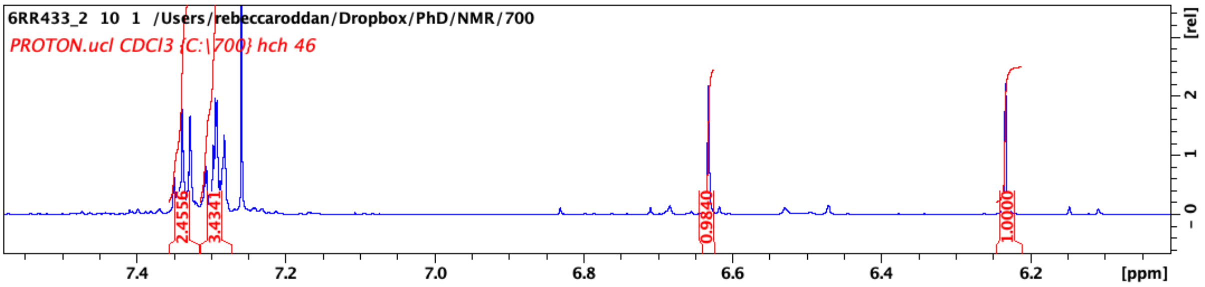

^{1}H-NMR (700 MHz): 4.0 – 2.7 ppm

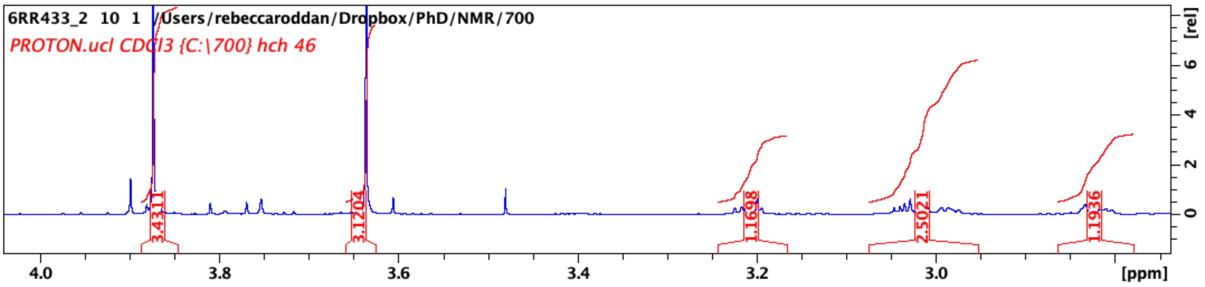

^{13}C-NMR (176 MHz)

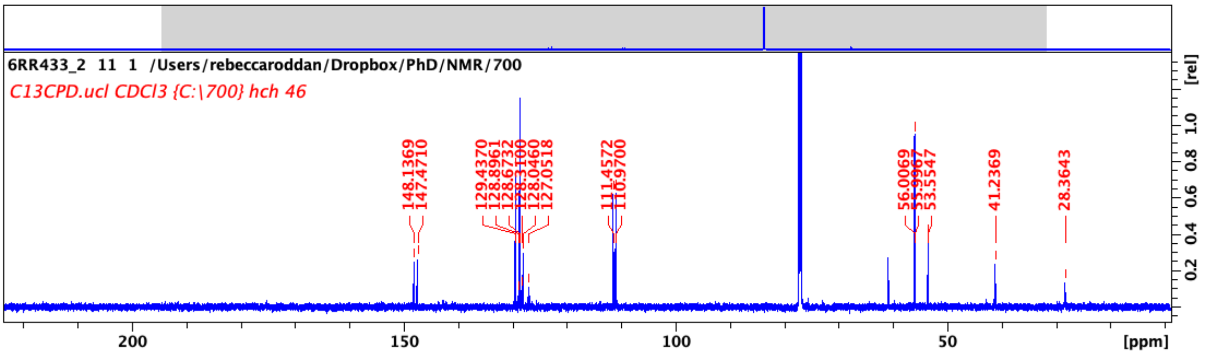

LCMS data of reactions with 4a

pQR2595 (4a and 4b)

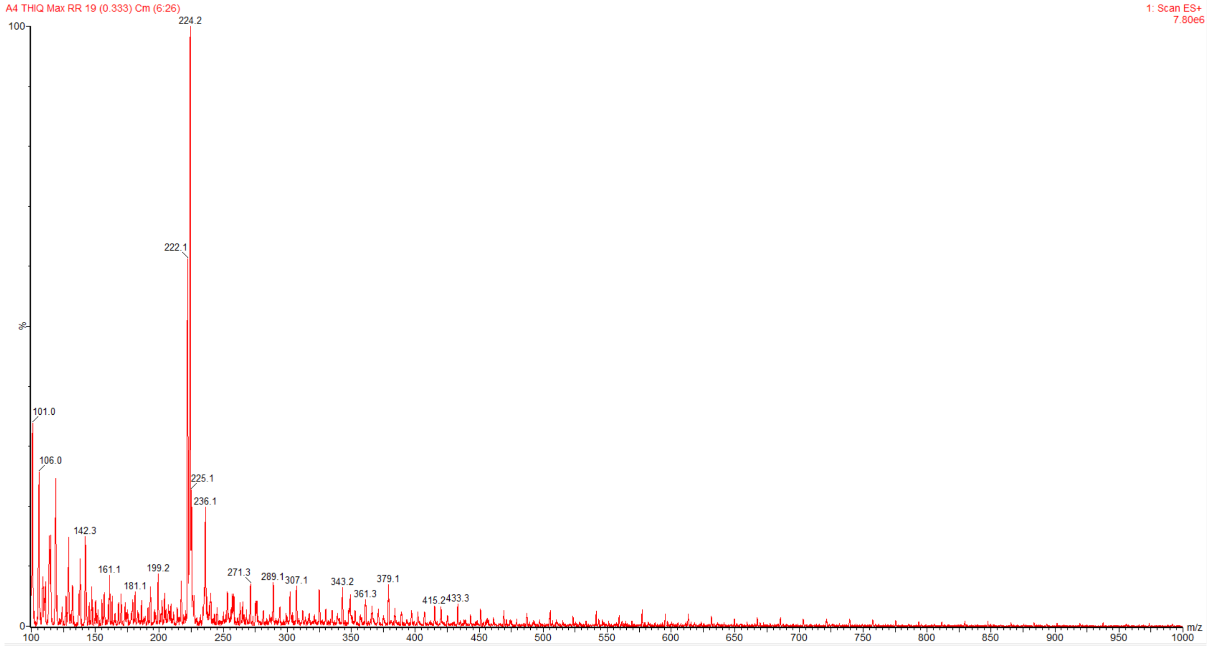

pQR2598 (4a and 4b)

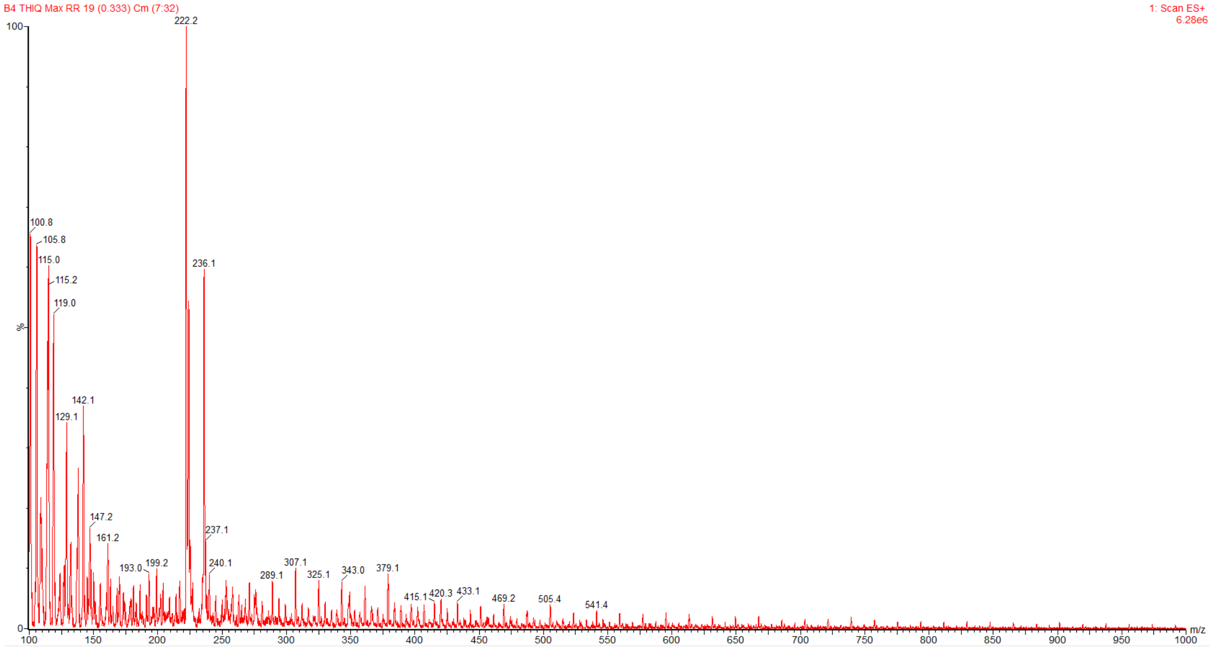

pQR2600 (4a and 4b)

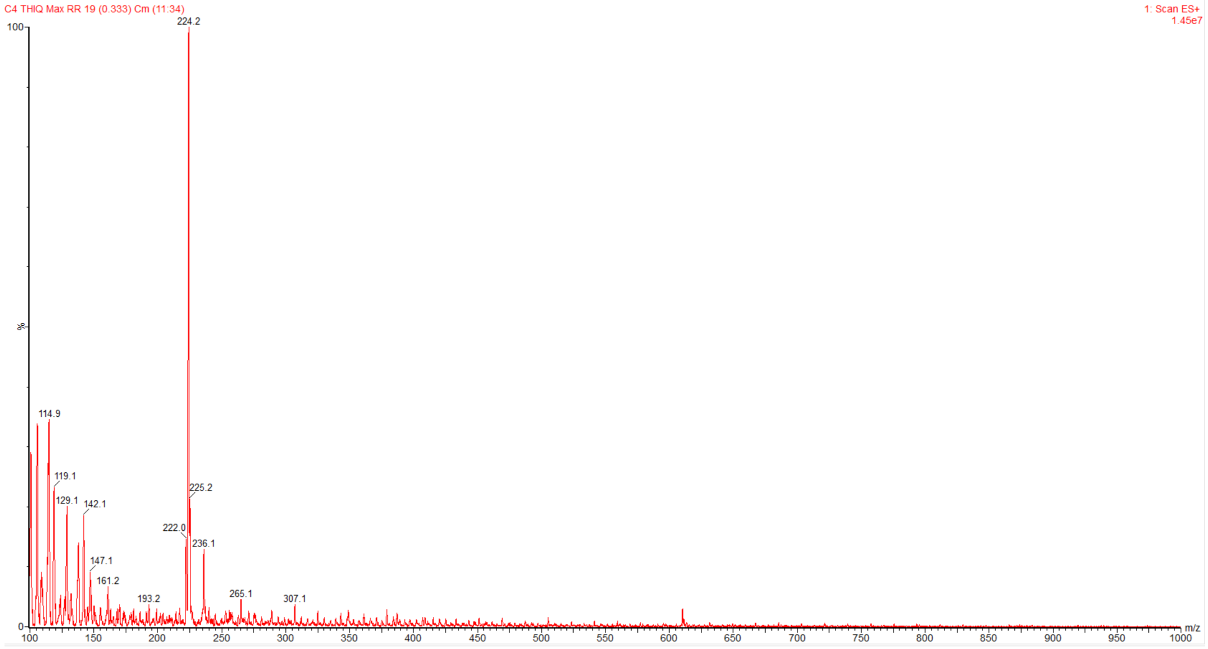

pQR2601 (4a and 4b)

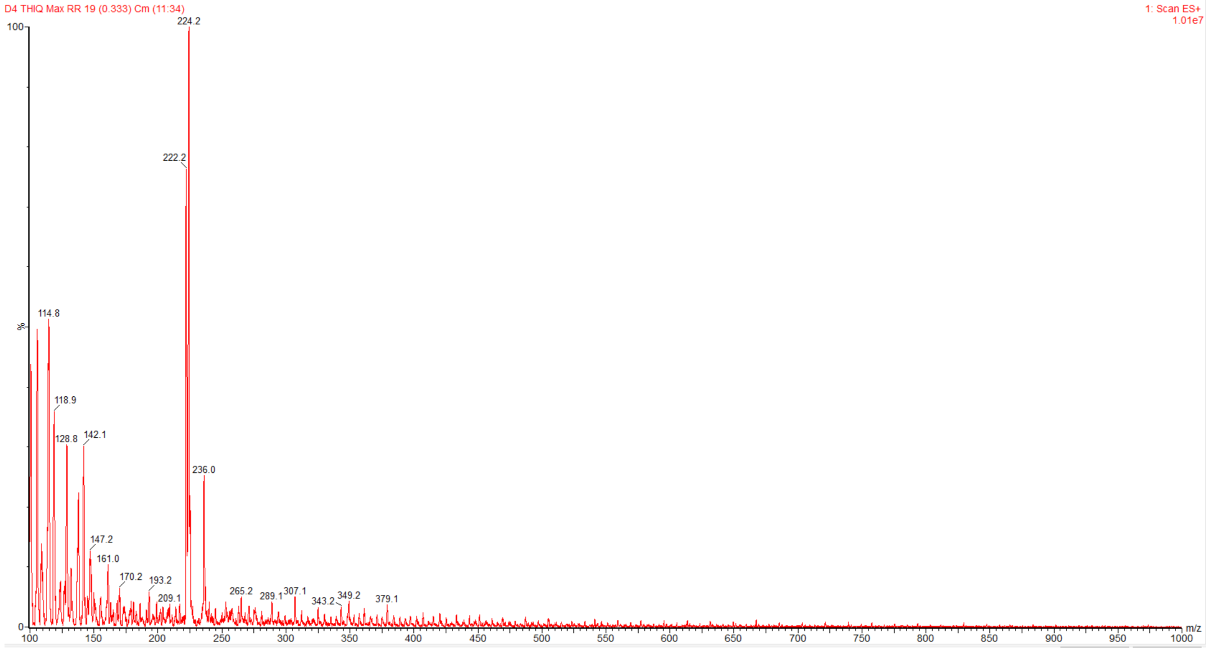

pQR2614 (4a and 4b)

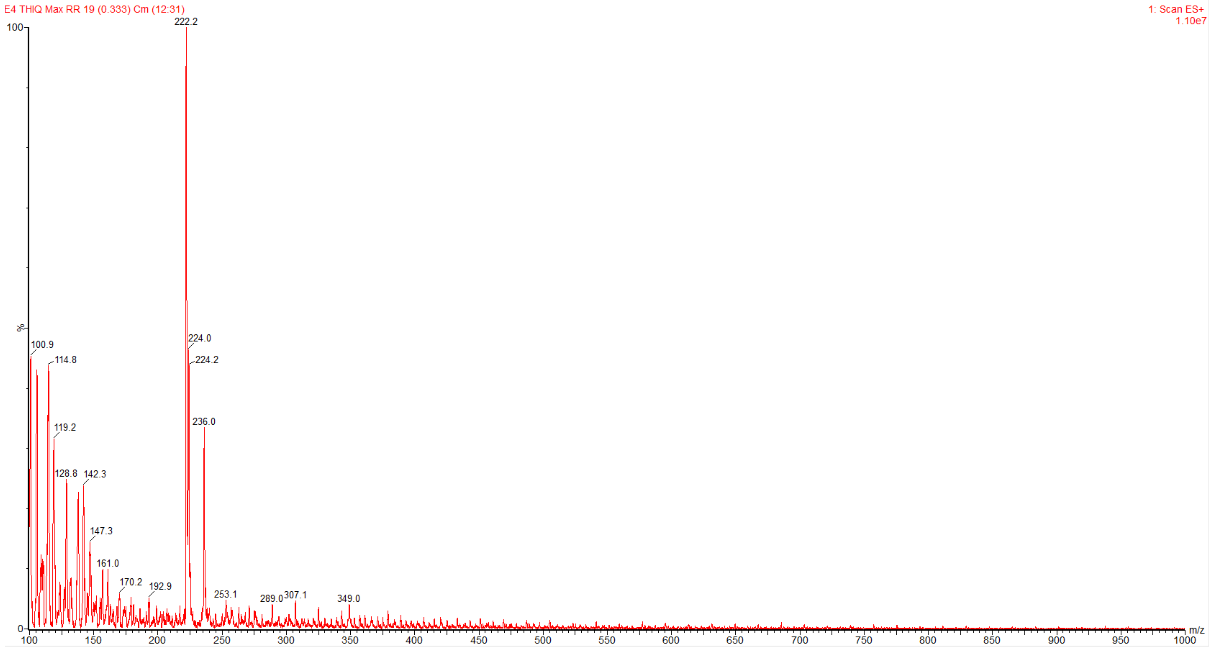

References

[1] H.-T. Luu, S. Wiesler, G. Frey, J. Streuff, *Org. Lett.* **2015**, *17*, 2478–2481.

[2] M. Ružič, A. Pečavar, D. Prudič, D. Kralj, C. Scriban, A. Zanotti-Gerosa, *Org. Process Res. Dev.* **2012**, *16*, 1293–1300.

[3] M. Perez, Z. Wu, M. Scalone, T. Ayad, V. Ratovelomanana-Vidal, *European J. Org. Chem.* **2015**, 6503–6514.

[4] N. M. Gray, B. K. Cheng, S. J. Mick, C. M. Lair, P. C. Contreras, *J. Med. Chem.* **1989**, *32*, 1242–1248.
